# The initial interplay between HIV and mucosal innate immunity

**DOI:** 10.3389/fimmu.2023.1104423

**Published:** 2023-01-30

**Authors:** Valeria Caputo, Martina Libera, Sofia Sisti, Benedetta Giuliani, Roberta A. Diotti, Elena Criscuolo

**Affiliations:** Laboratory of Microbiology and Virology, Vita-Salute San Raffaele University, Milan, Italy

**Keywords:** HIV, innate immunity, microbiome, sexual transmitted disease, mucosae

## Abstract

Human Immunodeficiency Virus (HIV) is still one of the major global health issues, and despite significant efforts that have been put into studying the pathogenesis of HIV infection, several aspects need to be clarified, including how innate immunity acts in different anatomical compartments. Given the nature of HIV as a sexually transmitted disease, one of the aspects that demands particular attention is the mucosal innate immune response. Given this scenario, we focused our attention on the interplay between HIV and mucosal innate response: the different mucosae act as a physical barrier, whose integrity can be compromised by the infection, and the virus-cell interaction induces the innate immune response. In addition, we explored the role of the mucosal microbiota in facilitating or preventing HIV infection and highlighted how its changes could influence the development of several opportunistic infections. Although recent progress, a proper characterization of mucosal innate immune response and microbiota is still missing, and further studies are needed to understand how they can be helpful for the formulation of an effective vaccine.

## 1 Introduction

The Joint United Nations Programme on HIV/AIDS (UNAIDS) has established to aim the 95-95-95 target (95% of all HIV-positive patients to know their HIV status, 95% of all diagnosed HIV-positive patients to receive therapy, and 95% of all treated HIV-positive patients to have viral suppression) by 2025, to reach the more ambitious goal to stop to consider AIDS as a public health concern in 2030 (1). However, the current numbers of HIV infections remain substantial, with 38.4 million people living with HIV and 1.5 million new cases in 2021 (2). Global use of antiretroviral therapy (ART), increasing prevention, and surveillance are being employed to contain the epidemic, but an effective vaccine is needed to end it.

Over the years, significant efforts have been made to study the pathogenesis of HIV infection; however, several aspects need to be clarified, including how the innate immune system acts in different anatomical compartments. Despite the molecular mechanisms were not completely understood, the importance of innate response in the progression of HIV infection was demonstrated by many studies on HIV-infected long-term non-progressors (LTNPs) and elite controllers (ECs), a particular group of HIV patients able to naturally control the viral replication. In fact, it was shown that the high level of cytokines, as IL-12 and IFN-α, and the increasing number and activity of innate cells, in particular dendritic cells (DCs), natural killer cells (NKs), macrophages and natural killer T cells (NKTs), and invariant natural killer T cells (iNKTs), can control the infection and delay AIDS progression (3–5). In recent years, extensive studies have been conducted on unconventional or pre-set T cells, such as mucosal-associated invariant T (MAIT) cells and iNKTs that can recognize several viral, bacterial, and cancer-associated epitopes and act as immunomodulators, due to their importance in mucosal immunity. During the early stage of HIV infection, IL-17-producing CD8+ MAIT cells decreased significantly, compromising the mucosal integrity and facilitating microbial translocation. The high activation, the consequent exhaustion and the depletion of these cells lasted a long time in the bloodstream, even during ART (6). Early after acute HIV infection, iNKT depletion has also been reported in the peripheral blood and is associated with markers of infection progression (7). On the other hand, the enrichment of cytokine-activated MAIT in the intestinal mucosa was described as a defense mechanism against the rapid depletion of other lymphocytes and the excessive activation of MAIT enhances inflammation and facilitates disease progression (6, 8, 9). Also, at the mucosal level, the importance of innate immunity in preventing HIV infection is demonstrated by the low-risk transmission levels, ranging from 1.38% of transmission through the rectal route to almost zero for the oral one (numbers estimated for exposure act) (**Table 1**) (16). Nevertheless, in 2020, male-to-male sexual contact counted for 68% of all new cases in the United States, followed by 22% of all HIV diagnoses caused by heterosexual contact (17). These numbers highlight the importance of transmission through these routes. Thus, understanding the molecular mechanisms and pathways that cause and follow infection is also pivotal in developing an effective therapeutic and prophylactic treatment that could prevent mucosal transmission.

**Table 1:** Risk of HIV transmission by mucosal/sexual exposure (no condom use).

Exposure act	Risk per exposure (%)	References
Receptive anal intercourse	1.38	(10–13)
Insertive anal intercourse	0.11	(10, 12)
Receptive penile-vaginal intercourse	0.08	(14)
Insertive penile-vaginal intercourse	0.04	(14)
Receptive oral sex	Low	(10, 15)
Insertive oral sex	Low	(15)

HIV is a retrovirus of approximately 100 nm surrounded by a lipid membrane as its envelope. The envelope contains 72 trimers of the Env proteins, gp120, and gp41. Inside the envelope, a conical capsid contains two copies of positive-sense single-stranded RNA that encodes structural and regulatory proteins. The first open reading frame is the *gag* gene, which codes the proteins of the outer core membrane, p17, the capsid protein, p24, the nucleocapsid, p7, and a smaller protein. The second is the *pol* reading frame, which encodes for the protease, p12, reverse transcriptase, p51, RNase and integrase, p32, while the *env* reading frame encodes for gp120 and gp41. Moreover, the HIV genome encodes for several regulatory proteins: Tat (transactivator protein) and Rev (RNA splicing regulator) are necessary for viral replication, while the other four (Nef, Vif, Vpr, and Vpu) are accessories for viral replication, budding, and pathogenesis (18, 19).

The Env proteins are the first viral proteins involved in the replication cycle; gp120 and gp41 mediate the binding to the cell, and the fusion to the cellular membrane, respectively (20). In particular, the binding of gp120 to the receptor CD4 and a coreceptor – CCR5 or CXCR4 – induces a series of refolding events in gp41 that enable the fusion to the target cell membrane (20). In addition to these receptors, HIV exploits the presence of other attachment factors, like integrins, glycolipids, and proteoglycans, allowing HIV to enter in several cell types. HIV mainly infects T cells, monocytes, macrophages, and dendritic cells (DCs), but also transcytosis and trans-infection were described in epithelial cells and fibroblasts, respectively (21–23). In permissive cells, the virus undergoes transcription, genome integration, replication, and budding from the cellular membrane. During the viral replication cycle, numerous nucleic acid-derived pathogen-associated molecular patterns (PAMPs) can be recognized by pathogen-recognition receptors (PRRs) of the host. The sensing of HIV infection leads to the activation of a cell-intrinsic innate immune response against viral infection (24) starting with macrophages and dendritic cells and progressing to the activation of natural killer cells (NKs) (25).

We focused on the innate immune component elicited within each mucosal tissue affected by sexually transmitted HIV. Starting from the physical barriers given by the mucosa tissue, we discussed how the integrity of these barriers is lost during the infection, and how cell-virus interaction leads to the activation of the cellular components of the innate immune response. Studying the events that occur within the first 96 hours after infection (26) is not easy, so the need for new discoveries that investigate unsolved aspects is tangible. Moreover, recent studies reveal the enrichment of *Prevotella* in the feces of the elite controllers (27), and the correlation between gut dysbiosis and HIV infection was so strong to suggest fecal microbiota transplantation as a possible therapeutic treatment to improve the HIV-positive patients’ status (5). We also discussed the engagement of mucosae’s microbiota and how its composition changes after HIV infection, but also how its impairment causes the development of opportunistic infections that characterized the clinical picture of many HIV patients.

## 2 The structure of mucosae as a physical barrier

The mucosal epithelium assumes specific characteristics in relation to anatomical district and function within the single mucosa. Overall, the oral and anogenital mucosae show specific structural characteristics that create a strong first-line defense against HIV transmission. The stratified squamous epithelium, which largely makes up the mucosae, is a concrete physical barrier that separates the lower tissues from the external environment. This structure, along with the presence of numerous tight and adherent junctions involved in maintaining tissue functionality, are essential elements in preventing HIV transmission. Both types of epithelial junctions form highly efficient biological barriers to paracellular entry by viral pathogens, including HIV (28).

The oral mucosa presents strategic levels of keratinization in the different layers of squamous epithelial cells with varying thicknesses (29, 30). Structures subjected to mechanical forces (i.e., the gingiva and hard palate) composing the masticatory mucosa are protected by a thick keratin layer that makes antigen entry difficult (29). Areas comprising mobile structures or specialized structures, such as lingual papillae, have a non-keratinized composition or a mixture of keratinized and non-keratinized epithelia (31). The thickness of the adult oral mucosa was demonstrated to be directly involved in reducing HIV transmission efficiency, also because of the absence of HIV-susceptible immune cells in the superficial mucosal layers (32).

In the female genital tract (FGT), instead, the epithelium varies between the lower and upper reproductive tract. The lower portion, comprehending the vagina and the ectocervix, is characterized by a pluristratified epithelium in which the continuous sloughing of the superficial layers of epithelial cells prevents many pathogens from colonizing it. In contrast, the endocervix, the endometrium, and the fallopian tubes of the upper reproductive tract are characterized by a single layer of polarized, columnar epithelial cells with tight junctions that prevent pathogens from breaching the epithelium (10, 33). The transition area from columnar to squamous epithelial is called the transformation zone (34). This area is composed of metaplastic cells, which represent the reservoir of the endocervix (35). In addition to the morphological characteristics, the transformation zone is the most immunologically active site in the reproductive tract, and is an efficient barrier against ascending pathogens (35). At the same time, the high levels of macrophages, CD4^+^, and CD8^+^ lymphocytes may suggest that this site is particularly vulnerable to HIV infection (35).

The male genital tract (MGT) consists of two major parts: the penile urethra and the testes. In uncircumcised males, the foreskin provides both physical and immunological protection to the glans (36). The adult foreskin is a double layer of skin – outer and inner – that covers the glans penis. The outer foreskin is a keratinized squamous epithelium, which constitutes a physical barrier to HIV infection. On the contrary, a thin and weakly keratinized epithelium composes the inner foreskin, making this site more susceptible to viral invasion (37).

Moreover, the anorectal mucosa is divided into two structurally distinct tissues. The lower canal (anal tissue) is lined with stratified squamous type II epithelium, while the upper part (rectal tissue) is composed of a single layer of columnar epithelium, making it more prone to mechanical trauma and viral infection during sexual intercourse through micro abrasions and transcytosis across epithelial cells (38). As with the FGT, the margin where the two different epithelia join is referred to as transformation zone. This area is most exposed to micro abrasions during receptive intercourse and to the chemical factors of microbiological organisms (39), making it the most accessible to HIV infection.

As observed in rectal tissue, variations in the structural and functional integrity of the mucosae represent the main factors exploited by the virus to establish the infection. Altered mucosal structure during sexual intercourse promotes HIV infection, particularly in the vaginal wall and the ectocervix (40).

Increased risk of HIV transmission is also reported in different scenarios characterized by damaged or inflamed oral mucosal tissues (41). Under these conditions, the transient migration of inflammatory cells from the deeper layers of the oral epithelium is facilitated. The recruitment of immune cells, including HIV-targeted cells, in the oral cavity, leads to a concrete increase in viral infection.

## 3 Oral mucosa

In adults, only a small fraction of new HIV infections is transmitted orally. Despite the viral loads reported for semen and cervicovaginal fluids (CVF), the presence of well-developed innate immune mechanisms in the oral cavity makes the risk of HIV transmission after oral-genital contact during sexual intercourse extremely low (42).

### 3.1 Oral route for HIV transmission

Several events involved in HIV transmission *via* the oral cavity are still to be understood; for example, the role of the oral epithelium in the establishment of viral infection has represented an interesting element of investigation. It was shown that the inoculation of the chimeric simian/human immunodeficiency virus (SHIV) to the surface of intact oropharyngeal epithelia can lead to systemic infection of HIV-susceptible immune cells (43) but the primary site of infection is unknown.

The oral epithelium is the first site of HIV exposure. However, epithelial cells do not express the canonical virus receptor CD4, while levels of CXCR4 and CCR5 appear to be very low or undetectable (44–46). Oral keratinocytes can internalize HIV, but there is still no evidence that they can support viral infection and replication (45). In this context, HIV virions are more likely to migrate across mucosal epithelial cells, without infecting them, to reach susceptible CD4^+^ lymphocytes naturally integrated into or underlying the epithelial layer (47). Structural and functional abnormalities of the oral mucosa surface could have a profound impact on its susceptibility to HIV infection.

Oral epithelial cells play an important role in promoting viral transmission across the mucosal tissue. Indeed, they express on their surface a significant variety of non-canonical receptors, in this way, the virus is transferred directly to their target cells present in oral mucosal tissue (47). Among the different non-canonical receptors that are bound by the viral protein gp120, there are DC-SIGN (dendritic cell-specific intercellular adhesion molecule-3-grabbing non-integrin) (48), GalCer (glycosphingolipid galactosylceramide) (49, 50) and heparin sulfate glycans (HSPGs) (51). Both GalCer and HSPGs have been shown to be highly expressed on oral epithelial cells (52); conversely, the DC-SIGN receptor is highly expressed on mucosal Langherans/dendritic cells (LC/DC), enabling HIV to be captured also by intraepithelial and/or subepithelial LC/DC from the mucosal surface (53). Thus, HIV bound on the dendritic cells can migrate through the epithelium promoting the infection of CD4^+^ cells (54). Despite this, the density of LC/DC on oral mucosa is significantly lower than on vaginal, cervical, and foreskin mucosa, which may result in a lower risk of virus acquisition across it (55). Currently, there is no evidence that any of the non-canonical receptors can allow the infection of epithelial cells in a CD4/CCR5/CXCR4-independent way, underlying the importance of the role of these cells only in the establishment of initial contact events of the virus in the oral cavity (47).

The specific interactions of HIV with its receptors on oral epithelial cells play a key role in triggering the activation of multiple signaling pathways which results in the disruption of epithelial junctions (28). For example, it was demonstrated that HIV gp120 binding with GalCer, CXCR4, and CCR5 increases intracellular calcium leading to the activation of mitogen-activated protein kinase (MAPK) signaling. This event causes the disruption of epithelial and endothelial junctions by reducing the expression of tight junction proteins ZO-1, occluding, claudins 1, 3, and 4 (56, 57). Moreover, the viral Tat protein is directly involved in the aberrant internalization of tight junction proteins and their down-regulation and/or proteasome-mediated degradation (56). HIV interaction with oral epithelial cells determines the production and the release of proinflammatory cytokines that can activate the apoptotic pathway leading to metalloproteases (MMPs) and/or caspase-mediated degradation of junctional proteins (28). HIV-associated disruption of epithelial junctions allows paracellular penetration of virions that can reach HIV-susceptible cells into the epithelium for the establishment of systemic infection (52, 58). In this context, the lack of functional junctions between cells promotes the penetration and dissemination of other viral pathogens.

Infiltration of HIV-infected CD4^+^ T cells and LC/DC into the oral mucosa induces the secretion of virions leading to the activation of the aforementioned intracellular pathways. This results in the worsening of the barrier integrity conditions of mucosal epithelium, facilitating bacterial translocation and penetration of their metabolites in the tissue with the further activation of HIV-associated inflammation and disease progression (28).

Viral transcytosis, the transcellular transport of virions by vesicular/endosomal machinery of epithelial cells, has been also proposed as one of the possible pathways involved in HIV transmission through the mucosal epithelium. Transepithelial transcytosis was shown to occur in the epithelial cells of different tissues, including the oral mucosa. However, cell-free HIV transcytosis was demonstrated to be less efficient, with only 0.01-0.05% of virions from the initial inoculum that can translocate across oral epithelial cells (58–60).

### 3.2 Innate immune response

The characterization of the exact immune events right after HIV infection is very difficult considering the identification of individuals in the specific period preceding systemic virus dissemination and the establishment of the latent reservoir. Innate immunity offers efficient responses for the prevention of HIV transmission at the level of the oral mucosa, although many aspects of the specific responses implemented at this site are still undefined.

**Figure 1:**
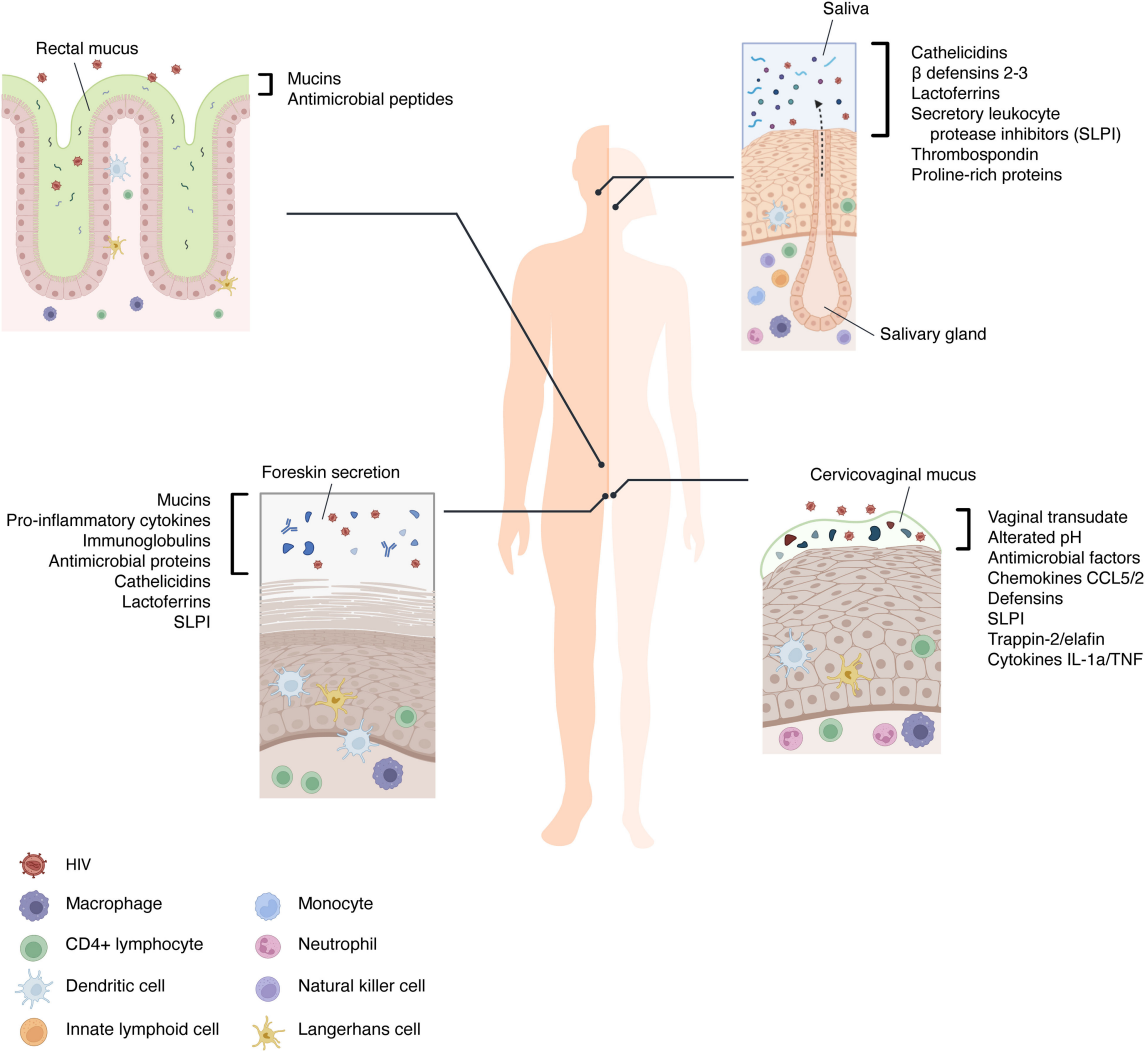
The immune components of mucosal barriers involved in HIV transmission. The extracellular environments of the oral, rectal, vaginal, and foreskin mucosae are diverse in their tissue architecture and characterized by numerous secreted factors that can hinder the mucosal transmission of HIV. In addition, saliva, foreskin secretion, and mucus are barrier-trapping and inactivating virions. Between the cells of the stratified epithelia and at their basal layers, the immune cells are present and recruited when HIV bypasses the first mechanism of defense.

A pivotal role in the low incidence associated with the oral route of transmission is attributed both to the presence of multiple anti-HIV salivary factors (**Figure 1**) and to the nature of human saliva itself (61). Daily secretion of saliva helps to physically eliminate pathogenic bacteria, viruses, and their products from the oral cavity (62). Moreover, it has been demonstrated that the hypotonic nature of this body fluid works as an innate antiviral factor causing the lysis of HIV-infected cells (63). In the context of sexual transmission, however, isotonic secretions such as semen allow overcoming this protective mechanism maintaining the viability of HIV-infected leukocytes that could initiate HIV infection of the oral mucosa (64).

**Figure 2:**
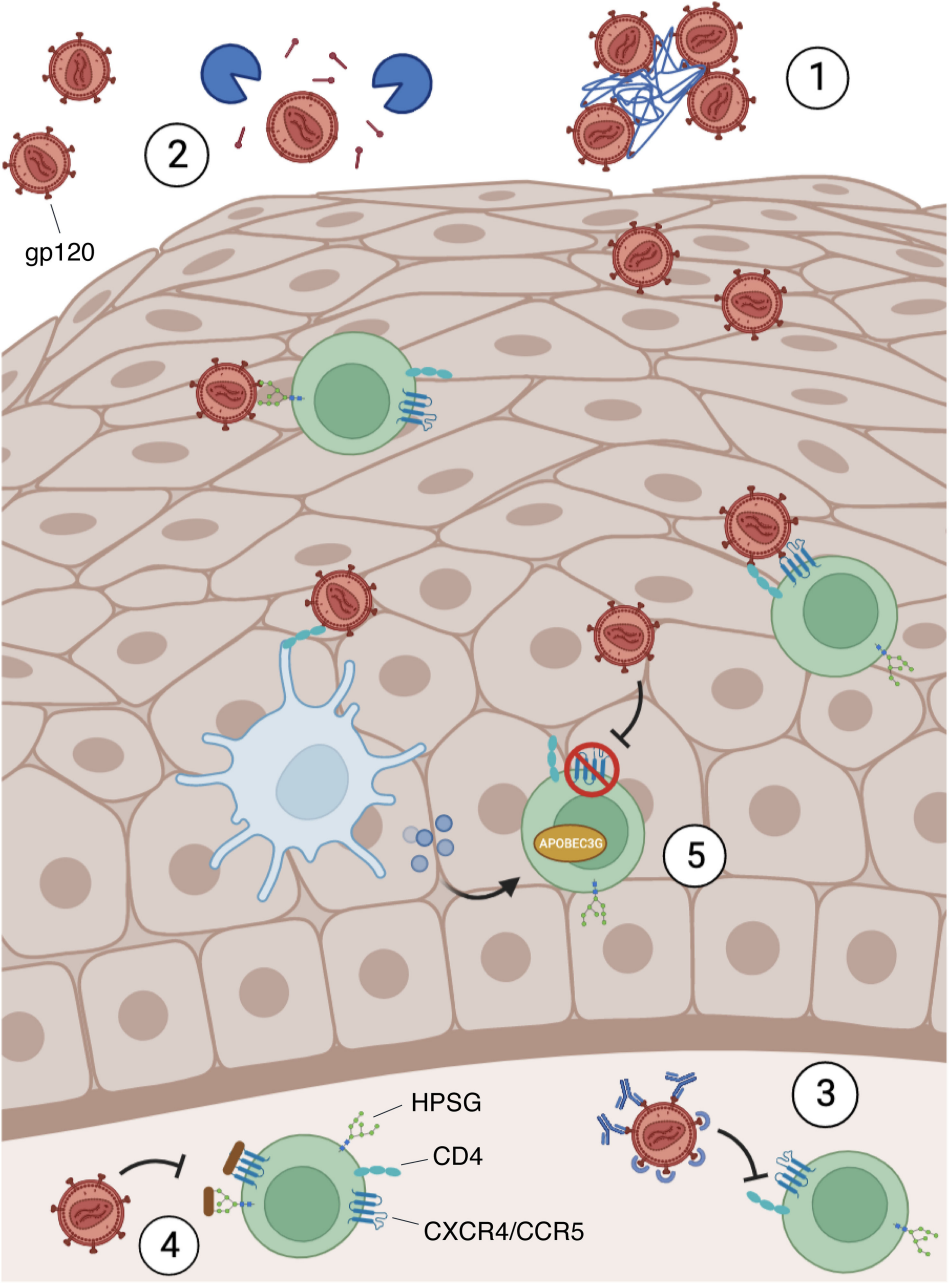
Blocking mechanism of HIV infection in the mucosae. HIV requires the binding between the envelope protein gp120, the cellular receptor CD4, and other co-receptors, such as CXCR4, CCR5, and heparan sulfate proteoglycans (HPSG). However, the mucosal microenvironment may present some soluble factors that can block the infection by several mechanisms: 1) Mucins interact with the virions, causing their agglutination; 2) Agglutinins and mucins can strip gp120 from the viral envelope; 3) immunoglobulins, thrombospondin and proline-rich proteins bind gp120 preventing its interaction with CD4 on target cells; 4) Defensins and lactoferrin recognize HPSG and HIV co-receptors (CXCR4/CCR5) on the surface of susceptible cells and compete with gp120 for their binding; 5) Defensins and IFNs secreted by stimulated immune cells (e.g. Dendritic cells) led to CXCR4 downregulation and APOBEC3G induction, an antiviral restriction factor.

As mentioned above, saliva contains many molecules which are directly released from resident cells and from different glands housed in the oral cavity. Among these, mucin-rich fractions of submandibular/sublingual saliva cause HIV particles to aggregate with a subsequent reduction of viral infectivity (65). Salivary mucins and agglutinins can also interact with viral particles inducing the stripping of HIV gp120 from the envelope: this causes a decrease in viral infectivity (66). Other specific salivary proteins that appear to inhibit HIV infectivity by direct interaction with the virus include thrombospondin and proline-rich proteins. These proteins can bind gp120, preventing its interaction with the CD4 receptor on target cells (67, 68) (**Figure 2**).

Furthermore, lactoferrin (LF) can strongly interact with the V3 domain of HIV gp120, thereby inhibiting virus entry (69). However, this glycoprotein is thought to carry out its primary biological activities following the interaction with its receptors, which include CXCR4 and HSPGs, interfering with the virus-cell fusion and binding. For instance, the membrane-penetrating HIV Tat protein, released from HIV-infected cells, also uses HSPGs to surround and enter cells: the binding ability of LF allows it to compete with such viral protein for receptor occupancy (70).

Other salivary molecules such as human β-defensins (hBDs) and cathelicidins inhibit viral replication. In particular, hBD2 and hBD3 can inhibit viral replication in primary human PBMCs and CD4^+^ cells (71). In this context, it was demonstrated that hBD2 inhibits at an early-stage post-entry, involving the induction of the host antiviral restriction factor apolipoprotein-like 3G (APOBEC3G) exploiting the chemokine receptor 6 (CCR6) which is expressed on highly permissive cells for HIV infection (72, 73). hBD2 and hBD3 exert their mechanisms directly on HIV virions and promote the downregulation of CXCR4 (71, 74). These hBDs also compete with viral gp120 for HSPGs binding on host cells (75).

The C-terminal part of human cathelicidins called LL-37 (or hCAP18) can be cleaved *in vivo* into active fragments: one of these fragments, called FK-13, inhibits HIV replication in peripheral blood mononuclear cells (PBMCs) (76). Moreover, *in vitro* studies have demonstrated that LL-37 has inhibitory efficacy against HIV infection in HEK293 (77) and primary CD4^+^ T cells (78). The study of the potential anti-HIV activity of LL-37 and its fragments demonstrated that this molecule could inhibit dose-dependently viral reverse transcriptase and, in weak measure, also virus protease (79).

Another significant HIV inhibitory molecule commonly found in saliva but also in semen and cervical secretions is represented by secretory leukocyte protease inhibitor (SLPI) (80). It has been shown to be present in saliva at levels sufficient to effectively inhibit the infectivity of HIV *in vitro* (81). SLPI does not appear to exert its antiviral activity directly on HIV virions but rather targets molecules expressed on host cells. Preincubation of monocytes alone with SLPI produced antiviral activity, whereas preincubation of the virus with this protein did not suppress or inhibit HIV virions (81). Afterward, it was established that SLPI binds monocytes with high affinity to a single class of receptors in a dose-, pH-, and time-dependent manner (82). Analysis of the newly generated viral DNA showed that SLPI blocks at or before viral DNA synthesis. Therefore, it was proposed that SLPI inhibits a step of infection that occurs after virus binding before reverse transcription (82). Depletion of SLPI from whole saliva results in a substantial loss of salivary anti-HIV activity (61). Higher levels of salivary SLPI in infants reduced the risk of HIV infection; however, breast milk levels of SLPI seem not to correlate with reduced breastfeeding transmission of HIV (83, 84).

In addition to the production of anti-HIV salivary secretory component investigated, other specific roles of innate immune cells in protecting the oral mucosa from HIV infection are much less characterized.

Oral epithelial cells are not only a mechanical barrier, but they are among the main sources of pro-inflammatory and regulatory cytokines released into the saliva during oral infections. A broad range of cytokines is also produced by other resident cells of the oral mucosa such as macrophages, fibroblasts, mast cells, and intra-epithelial lymphocytes (62). Differences in the saliva cytokine profiles of HIV-infected subjects compared with non-HIV-infected subjects were evidenced by a significant decrease of tumor necrosis factor (TNF) -α and interleukin (IL) -6 and a significant increase of IL-8 in HIV infection. In particular, IL-8 levels were also different among HIV-infected individuals on and off ART (85).

The animal model for simian immunodeficiency virus (SIV) infection shows the presence of NK cells in the tonsillar, buccal tissues, and oral-draining lymph nodes consequently producing large quantities of interferon (IFN) -γ and the β -chemokine MIP-1β (86). Active SIV replication in oral mucosa also induces the upregulation of granzymes and other NK biomarkers such as CD16, NKG2C and relative KIR expression, all indicative of a robust NK cell response (87).

The characterization of innate lymphoid cells (ILCs) has also generated interest. This is a subpopulation of mucosa-restricted cells with features similar to both NK cells and Th17 and Th22 (88, 89). SIV-infected macaques present an expansion of ILCs in oral-draining lymph nodes and tonsils, producing large amounts of IL-17 and TNF-α (86). This contrasts with the scenario observed in the gut, where massive depletion of NK cells and ILCs was observed (90).

An active role in preventing HIV infection was also identified for polymorphonuclear neutrophils (PMNs). The function of PMNs in HIV disease has mainly been examined from the point of view of patients’ increased susceptibility to opportunistic infections. Dysbiotic microbiota at the mucosal surface and inflammatory conditions (e.g., periodontal disease) can influence neutrophil recruitment and activation in oral tissues (91, 92). Whereas PMNs are directly involved in the production and release of defensins, and their activation can lead to the production of neutrophil extracellular traps (NETs) for HIV virions capture and elimination (92).

### 3.3 Oral microbiota

The oral microbiota can be affected by different factors, including diet, smoking, and drugs, but also changes in the saliva secretory component (such as reduced levels of enzymes and proteins) or alterations of the innate and adaptive immune response can be correlated to dysbiosis (93). Evidence suggests that HIV infection impacts the composition of the oral microbiota, despite inconsistent results.

**Figure 3:**
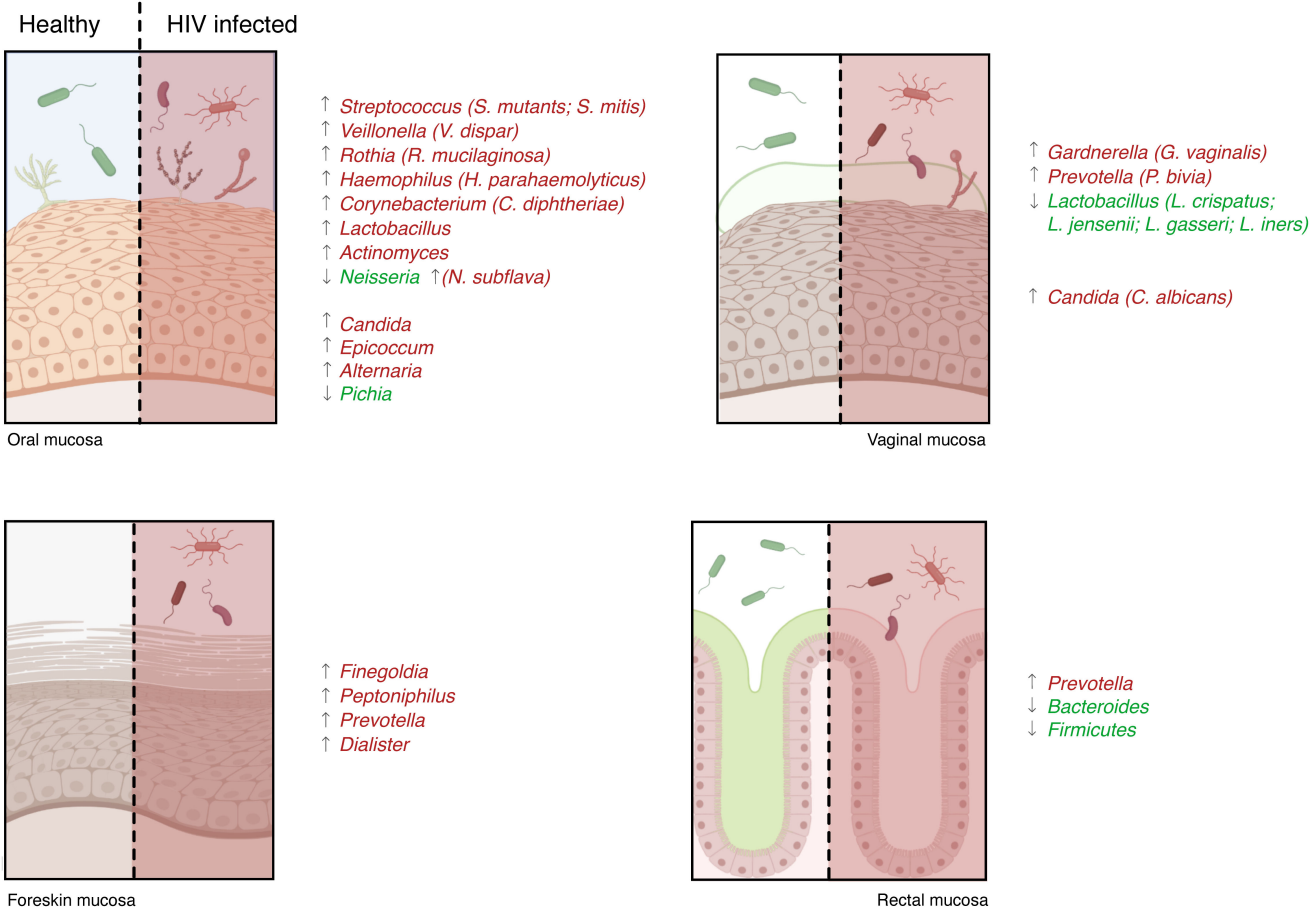
Microbiota characterization in HIV patients. The microbiota present in the mucosae can support or penalize HIV infection, and its changes can influence the development of several opportunistic infections. Numerous studies analyzing HIV-infected and -uninfected patients’ mucosal samples identified a different microbiota between the two populations: some species were higher in healthy controls (species reported in green) and others were more abundant during infection (species in red).

A recent study analyzing the saliva of HIV-infected and -uninfected patients found a different microbiota between the two populations. They found that the abundance of *Streptococcus* was increased in HIV-infected individuals, while the abundance of *Neisseria* was higher in healthy controls (94). Another study showed similar results: the abundance of *Neisseria* was decreased in HIV-infected patients while the abundance of *Veillonella, Rothia*, and *Streptococcus* increased significantly (95). Furthermore, *Streptococcus mutans, Lactobacillus, Candida, Haemophilus parahaemolyticus, Actinomyces, Neisseria subflav*a, and *Corynebacterium diphtheriae* species were more abundant in the saliva of infected HIV individuals (96–100). Other studies also observed a lower proportion of *Streptococcus mitis* in the saliva of HIV-infected patients compared to non-infected people (97, 98, 100) (**Figure 3**). In addition, alterations in the oral fungal community composition in HIV-infected patients were described,in particular, an abundance of *Candida, Epicoccum*, and *Alternaria*, and a decrease of *Pichia* were observed (101, 102). Therefore, given the anti-*Candida* activity of *Pichia* fungus, its absence leads to an increase in human infections, such as oral candidiasis, in HIV-infected patients (102, 103).

The disruption of oral immunity in HIV-infected individuals leads to an imbalance between oral microbiota and local immune responses, which could promote oral dysbiosis contributing to the development of HIV-related diseases and HIV-associated non-acquired immunodeficiency syndrome comorbidities (104). The most common comorbidity that occurs in HIV-infected patients, even in patients under ART therapy, are periodontal diseases, oropharyngeal candidiasis (OPC), oral warts, oral hairy leukoplakia, and Kaposi sarcoma (KS) (105). Of these infections, OPC is the most common (106) and it is caused by *Candida albicans* (107–109). A distinct oral microbiota may affect the development of oral diseases in HIV-infected patients, an example is an enrichment of *Abiotrophia, Rothia*, and unclassified *Pasteurellaceae* and *Treponema.* in the oral microbiota of HIV-infected individuals with moderate and severe periodontal disease (110), while HIV-infected individuals with oralKS presented a decrease of abundances of *Aggregatibacter* and *Lautropia* and an abundance of *Corynebacterium* and *Shuttleworthia* (111). It is also important to point out how HIV-infected patients are susceptible to a variety of other viral infections, which may accelerate the progression of HIV infection. The most common viruses identified in the oral mucosa and associated with oral lesions in HIV-infected patients are human herpesviruses (HHV-6 and HHV-8), cytomegalovirus (CMV), Epstein-Barr virus (EBV), varicella-zoster virus (VZV) and human papillomavirus (HPV) (112–115). In detail, HHV-6 and CMV can be considered cofactors for CD4^+^ cell decrease in HIV-infected patients promoting the progression to AIDS, and also coinfection with HHV-8 contributes to the pathogenesis of Kaposi’s sarcoma (116–119). Furthermore, in immunosuppressed patients, VZV tends to be reactivated and cause oral ulcers while co-infections with EBV are associated with the development of oral hairy leukoplakia (120, 121).

## 4 Genital mucosa

### 4.1 Female genital tract

The mucosal immunity in the female genital tract is part of the integrated mucosal immune system, with features related to specific reproductive functions. Indeed, the FGT immune system is closely regulated by cyclic changes in the sex hormones during the menstrual cycle, in a delicate balance between tolerance to allogenic sperm and semi-allogenic fetus and immune protection against infections (122, 123).

To establish infection in FGT, HIV in male ejaculate must first evade a number of intrinsic mechanical, chemical and biological barriers. FGT’s epithelial and immune cells express a repertoire of Toll-like receptors (TLRs) that enable them to recognize pathogens and mount a response to infections (124). Indeed, the activation of the TLRs response leads to the production of pro-inflammatory chemokines and cytokines (IL-6, IL-8, SDF-1), as well as to the recruitment of the resident immune cells (125). Unlike the lower FGT cells, which express a small list of TLRs (1-3, 5, and 6), the upper FGT is characterized by the expression of TLRs 1-9 (126). In addition, all the FGT cells also express nucleotide-binding oligomerization domain (NOD) receptors, which act as antimicrobial sensors (127). NODs are cytoplasmic pattern-recognition receptors, and their engagement results in NFκB activation and production of pro-inflammatory chemokines and cytokines, such as CXCL8 (128).

In the FGT, viral particles could penetrate the epithelium through transcytosis or paracellular passage. To reach the submucosal compartment, HIV could bind epithelial surface molecules, such as sulfate proteoglycans or GalCer, which induce the internalization of viral particles (60). The production of pro-inflammatory cytokines and IFNs induces a reorganization of the actin-cytoskeleton, and infectious virions can be released in the basal space, where they could encounter susceptible cells (129). Alternatively, viral-host receptor binding induces a reduction in the expression of occludins and claudins, thus causing a disruption of tight junctions and allowing paracellular passage (130). The distinct abundance of immune cells in the FGT tract may indicate a different susceptibility to HIV infection. The high concentration of immune cells in the lamina propria of the transformation zone suggests that this might be the primary infection site (35).

Nevertheless, there are other factors that can influence susceptibility to HIV infection. Fluctuations in estradiol and progesterone have resulted in a change in the innate and adaptive immune responses of FGT, suggesting an increased risk of sexually acquired infections (131). Hormonal changes during the menstrual cycle, contraceptive drugs, and menopause affect the thickness of the epithelial layer, influencing the permissiveness of HIV infection (132). HIV infection is more likely 7-10 days after ovulation – the “window of vulnerability” – because of the diminished protective immune response that is necessary to avoid activating the immune response against the semi-allogenic fetus (133). Moreover, different studies highlighted the presence of co-factors that could increase the risk to acquire HIV. Notwithstanding its important role in protection, mucosal inflammation of the FGT increases the probability of infection (134, 135). Under physiological conditions, the FGT environment contains less cytokines and immune cells. During inflammation, there is the recruitment of immune cells at the infection site, which are the target of the infection, and the production of cytokines, such as TNF and IL-1a, responsible for the disruption of epithelial barrier integrity (131, 136, 137). Multiple factors are involved in genital inflammations, such as hygiene, sexually transmitted diseases, and hormonal contraceptives (138, 139). Also, alterations in the microbiome composition could be considered a risk factor, promoting inflammation, and altering the correct pH of the mucus (140).

#### 4.1.1 Innate immune response

The structure of the epithelium, together with a thick layer of mucus that covers the lower FGT, acts as a physical and chemical barrier to prevent HIV from entering. Cervicovaginal mucus consists of vaginal transudate, mucus, antimicrobial factors, chemokines, and cytokines, including defensins, SLPI, trappin-2/elafin, which are associated with protection against HIV infection (141, 142) (**Figure 1**). Elafin and its precursor trappin-2 are members of the whey acidic protein (WAP) family that contain a characteristic and evolutionary conserved four-disulfide core, or WAP domain, which is rich in cysteine residues that stabilize the disulfide bonds involved in protease inhibition (143). *In vitro* studies revealed the critical protective role of the N-terminal portion of the elafin protein: it reduces IL-8 secretion and NF-kB activation and significantly modulated mRNA expression of innate sensors TLR3 and RIG-I in cervicovaginal fluids (143, 144).

The antigen-presenting cells in the FGT secrete multiple chemokines with antiviral actions that have been found in the cervicovaginal fluids, such as CCL5 and CCL2 (145). CCL5 has a pivotal role in attracting macrophages and dendritic cells at the site of infection, while CCL2 recruits inflammatory macrophages, as results from the analysis of the cervicovaginal lavage fluid of infected individuals (146).

FGT cells also produce IFNs, specifically IFN-α and IFN-β, which are well known for their potent innate immunity to broad-spectrum viruses (147). For example, IFN-α can significantly enhance the expression of APOBEC3G in CD4^+^ T cells derived from human peripheral blood mononuclear cells, inactivating the early stages of HIV infection (148) (**Figure 2**). In addition, the acid pH of the cervicovaginal mucus played a key role in trapping the virus, contributing to the protection from the infection (149). However, HIV virions exploit the presence of putrescine and cadaverine in semen to escape from acid inactivation and survive in the FGT environment (150).

Beyond the physical barrier and the antiviral factors, the cellular component of the innate immune system also responds to HIV infection. Neutrophils are the first responder to HIV infection, and once they are recruited, they engulf and destroy HIV through mechanisms like reactive oxygen species (ROS) release, degranulation, phagocytosis, and neutrophil extracellular trap formation (151). Despite evidence of the protective role of neutrophils in HIV acquisition, other studies suggest their role in promoting the infection. Indeed, neutrophils recruit CD4^+^ T cells in the FGT by secreting specific chemokines and cytokines, thus increasing the number of susceptible cells to HIV infection (152, 153).

One of the most important functions of mucosal dendritic cells is antigen presentation to the adaptive immune system, acting as a bridge between the innate and the adaptive response. The plethora of DCs subsets isolated from the FGT of healthy women could capture HIV virions and rapidly secrete CCR5 ligands and other chemokines, such as CCL2 and IL-8, which act as anti-HIV molecules (154). Although the primary target of HIV infection is CD4^+^ T cells, the level of FGT DCs, in particular Langerhans cells, seems to play a critical role in HIV transmission (155). The binding to DCs surface C-type lectin receptor, like DC-SIGN, allows the virions to migrate together with DCs to the lymphoid tissues, where HIV infects active CD4^+^ T cells, facilitating viral dissemination (155).

In addition, despite the presence of iNKT, HIV can be transmitted through viral immune escape strategies that block iNKT effector function against infected cells (156, 157).

#### 4.1.2 Vaginal microbiota

The vaginal microbiota plays a crucial role in reproductive health, including the potential to protect against HIV and sexually transmitted infections (STIs) and abnormal birth outcomes (158–160). Its composition is dependent on several factors including sexual activity, ethnicity, hygiene practices, antibiotics, the composition of the gastrointestinal microbiota, and the hormonal shifts associated with puberty, menopause, and the menstrual cycle (161–163). In general, the vaginal microbiota is characterized by a dominance of *Lactobacillus* species (particularly *L. crispatus, L. jensenii, L. gasseri*, and *L. iners* (164, 165) and changes in female genital tract microbiomes appear to influence the risk of HIV infection (164, 166).

The shift of vaginal microbiota composition from acidic-producing bacteria, *Lactobacillus*, to other anaerobic bacteria, especially *Gardnerella* and *Prevotella*, is associated with a clinical condition referred to as bacterial vaginosis (BV) (167, 168). Different studies reported that BV increases susceptibility to HIV infection (169). In particular, a study in Kenya showed that the presence of BV and the absence of *Lactobacilli* are significantly associated with HIV acquisition, highlighting that the presence of this bacterium seems to be protective not only against HIV infection, but also against the most common sexually transmitted infection (*Neisseria gonorrhoeae, Trichomonas vaginalis*, and *Chlamydia trachomatis*) (170, 171) (**Figure 3**).

Of particular interest, different studies highlighted how the presence of normal vaginal microbiota dominated by *Lactobacillus* had a protective role in the acquisition of HIV infections in high-risk HIV-negative women and a protective role in the transmission of the virus in HIV-positive women, reducing HIV shedding (172–174). This may be reinforced by a prospective cohort study of 236 South African adolescent girls not infected with HIV (18–23 years) (175). The authors pointed out how the girls with high-diversity, low *Lactobacillus* abundance bacterial communities were associated with a significantly higher risk of HIV acquisition compared to the girls with a high relative abundance of *L. crispatus* (175). Moreover, the detection of *L. crispatus* was also associated with a 35% lower risk of HIV RNA shedding bringing out its protective function in preventing HIV infection and transmission (172–174).

Additionally, other reports showed *Gardnerella vaginalis* and *Prevotella*. (specifically, *Prevotella bivia*) contributing to both HIV risk and genital inflammation (176, 177). A recent study conducted in Zambia on a cohort of pregnant women with and without HIV correlated the high prevalence of diverse, anaerobe-rich microbiota with HIV susceptibility and identified two *Gardnerella* spp. that may be associated with vaginal inflammation and with spontaneous preterm birth (sPTB) (178). Considering the important role of *Prevotella* bacteria in the BV inflammation state, several studies have identified a significant association between the abundance of *Prevotella* (*P. melaninogenica* and *P. bivia)* with increased genital inflammation and HIV acquisition (175–177). Furthermore, the above study conducted on healthy young South Africans identified that *P. bivia* and *P. melaninogenica* were significantly more abundant in girls with HIV than in girls without HIV infection (175).

Many studies have focused on the mechanisms by which the vaginal microbiota can affect HIV acquisition and transmission. Firstly, vaginal microbiota associated with BV can activate an immune response by recruiting mucosal immune cells and inducing pro-inflammatory chemokines and cytokines (175, 176, 179, 180). It was shown that BV is associated with the presence of an HIV-inducing factor (HIF) in vaginal secretions, which leads to increased virus replication in T cells and monocytes (181). Moreover, BV may be related to the disruption of the vaginal epithelium and the shedding of HIV to the subepithelium (182, 183). Lastly, the reduction in the number of *Lactobacillus* species causes an increase in pH and a reduction in H_2_O_2_ concentration, thus compromising the protection of the vaginal epithelium (184, 185). Another study performed in the USA comparing the vaginal microbiota of both HIV-infected and -uninfected women with or without BV observed that HIV-infected women with BV had higher microbial diversity which could be related to the suppression of immune response (186). Moreover, as mentioned above, the *G.vaginalis* and P*. bivia* could be involved in high HIV susceptibility risk and genital inflammation. In fact, *G. vaginalis* can produce several classes of cytolysin that activate the protein kinase pathway in vaginal epithelial cells, resulting in cell death (187). Similarly, *Prevotella* produces a specific enzyme able to degrade mucins, allowing microbial attachment and biofilm formation. *P. bivia* is the most common microbial profile among women infected with HIV and BV, and it is considered the most reliable predictor of both genital inflammation and HIV risk in women (188). An *in vitro* study confirmed that lipopolysaccharide (LPS) of vaginal *P. bivia* induced cytotoxicity, therefore, this anaerobic bacterium may contribute substantially to genital inflammation, which can influence barrier disruption and increase the risk of HIV in women with BV (189).

As for the other mucosae, HIV infection increases the likelihood of contracting other sexually transmitted- and opportunistic infections (190, 191). Several studies reported that HIV-positive women present a higher rate of vaginal *Candida* colonization often caused by *Candida albicans* (192–194), which increases over progressive immunodeficiency even if vaginal *Candida* colonization rates were 40% lower than those of oropharyngeal (195, 196). A study described a strong association between *Candida* infection and HIV seroconversion, highlighting the functional link between dysbiosis and HIV transmission risk. In fact, the initial HIV infection disrupts the local mucosal immunity, causing an alteration of the vaginal microbiome and *Candida* colonization (197). The inflammatory state caused by *C. albicans* compromised the integrity of the vaginal mucosa and increased the number of HIV target cells (leukocytes and other immune cells), enhancing HIV transmission during vaginal intercourse. This was also described in a study conducted on the vaginal microbiota of Tanzanian women, which reported that HIV-infected women had an abundance of *Candida* spp. and a severely compromised immunity in the lower genital tract (198).

Other sexually transmitted pathogens, *N. gonorrhoeae*, *Chlamydia trachomatis*, and *Trichomonas vaginalis*, are also common in HIV-seropositive women and are considered risk factors for HIV transmission. This underlines how the dysbiosis state caused by HIV infection can promote HIV transmission by activating an immune response to the genital region or by increasing the viral load in genital secretions (199–201). Moreover, clinical manifestations that occur in HIV-seropositive women are genital ulceration caused by *Treponema pallidum*, Herpes simplex virus (HSV), *Haemophilus ducreyi*, and CMV (202, 203). Genital herpes occurs more frequently in HIV-infected women than in uninfected women, which showed more severe clinical expression and asymptomatic viral shedding due to the disruption of the physical barriers of the skin (204, 205).

### 4.2 Male genital tract

Unlike the FGT, the mucosal immunity of the MGT is poorly assessed. Studies have shown that the foreskin secretion contains mucins, soluble mediators of the immune defense as pro-inflammatory cytokines and immunoglobulins, as well as antimicrobial proteins, which protect MGT from infections (37, 206) (**Figure 1**). For the cellular component, it has been described that the inner foreskin contains susceptible cells to HIV infection, including Langerhans cells and DCs, CD4^+^ T cells, and macrophages (207). There is a lack of understanding of how HIV is acquired in the foreskin. It is known that circumcision diminishes infection risk, reducing abrasions and ulcers (208).

#### 4.2.1 Male genital tract microbiota

The human penis is inhabited by diverse bacterial families and, just like the other mucosae, differs by abundance and type when the physiological condition changes. For example, it was demonstrated that circumcision affects bacterial profile, especially aerotolerance bacteria (209–211). In fact, the reduction in penile anaerobe bacteria may partly account for the reduced risk of heterosexually acquired HIV infection in men following circumcision (211).

To date, there are only two studies that analyzed the impact of the penile microbiota and HIV infection, and both assert that penile microbiota may be a risk factor for HIV infection in men (210, 212). They found that among men with and without HIV infection, there was a differential abundance of bacterial taxa (e.g., *Staphylococcus, Strenotrophominas, Propionibacterium*, and *Nosocomiicoccus*), thus suggesting that such bacteria either increase the risk of HIV infection or occur because of HIV infection (210). Moreover, they found that selected anaerobic bacteria such as *Finegoldia, Peptoniphilus, Prevotella*, and *Dialister* were associated with the increased risk of HIV seroconversion (211, 213) and elevated levels of chemokines, including IL-8 (212). This has already been reported in women who had high levels of IL-8 associated with an increased risk of HIV acquisition (135). Moreover, sexually transmitted infections such as *N. gonorrhoea* may also enhance HIV transmission by recruiting and activating HIV target cells at the site of primary infection (214) (**Figure 3**). Together, these investigations imply the involvement of penile microbiota (particularly of uncircumcised men) and immune activation responses in the acquisition and transmission of HIV (209, 211, 215). Penile bacteria can stimulate genital immune activation, increasing susceptibility to HIV infection (216).

As for HIV-infected women, also in HIV-infected men, the most common opportunistic infection that may occur in the genital tract is candidiasis and this infection can affect the head of the penis and the foreskin (217). HIV-infected patients are more susceptible to sexually transmitted bacterial pathogens, such as *Chlamydia trachomatis* and *Neisseria gonorrhoeae* (218), and to the reactivation and replication in the genital tract of persistent herpesviruses, such as CMV, EBV, and HSV-1 and -2 (219–221). HIV-HSV co-infection has been associated with increased HIV viral load, transmission, and progression of the disease (222, 223). Moreover, alterations of penile microbiota seem to be associated with HPV infections, in fact, it was demonstrated that men with penile microbiota dominated by *Prevotella, Clostridiales*, and *Porphyromonas* were more likely to have HPV infections than men with *Corynebacterium*-dominated penile microbiota (210).

## 5 Anorectal mucosa

The rectal mucosa is also a major mucosal HIV transmission route. The risk of transmission is approximately 1.38% per exposure act (224), and studies in rhesus macaques suggest that HIV acquisition by the receptive partner is higher during anal intercourse compared to vaginal intercourse (225). In fact, epidemiological studies report that over half of new infections in the United States are caused by receptive anal intercourse (RAI) (226).

As far as the current knowledge, the higher transmission rate compared to other mucosae is related to several key characteristics of the rectal mucosa that makes it more susceptible to HIV infection. First, as mentioned above, the mucosa has a peculiar structure (single layer of columnar epithelial cells) that makes the rectal tissue more prone to infection through mechanical trauma during sexual intercourse (38). Second, although less is known about cell-virus interactions in the rectum compared with other mucosal tissue, the presence of HIV-target CD4^+^ cells such as macrophages, dendritic cells, and T cells underneath the simple epithelium was described (227). In particular, the relative abundance of activated CD4^+^ T is very important for virus acquisition and mucosal transmission (228–230). Lastly, the more distal section of the rectum contains a greater concentration of CCR5-expressing macrophages (231).

Despite this, the events following exposure to HIV in the rectal mucosa and the initial targets for rectal infection remain unknown. A study reported findings consistent with mucosal injury (232, 233) indicating an upregulation (24h after) of gene transcription important in tissue remodeling, the involvement of neutrophils, DNA proliferation, and antigen presentation (233). The importance of neutrophils involvement has also been documented for both female and male genital tract, but their role in rectal transmission still needs further analysis (233). Regarding target engagement, some studies support macrophages as the first target (234, 235). One study observed a higher number of macrophages expressing CCR5 in the rectum than in the proximate colonic mucosa making this tissue more vulnerable to HIV infection (231). Macrophages can act as viral reservoirs disseminating the virus to T cells and DCs across mucosal tissue (236). Others identified a subclass of dendritic cells which express DC-SIGN in the rectum as capable of binding and transferring HIV virions to permissive T cells (48, 237). Lately, a study using an innovative methodology to infect rhesus macaque concluded that Th17 CD4^+^ T cells constituted the most abundant target, but also intestinal DCs (iDCs) as second target. These DCs are present at a low frequency in the anorectal tissue, but it is known that they express coreceptors CCR5 and receptor CD4, making them susceptible to infection. However, other cell types express these receptors and coreceptors at different levels, so other factors contribute to cell infection. Th17 cells and iDCs seem to express lower levels of Myxovirus resistance B (MxB), a restriction factor that inhibits HIV life cycle early phase, especially in type I IFN-influenced environment (238, 239). The higher metabolic state of these cells may also facilitate nuclear transport, integration, and transcription of HIV (240). On the contrary, as was already described, HIV does not replicate well in resting CD4^+^ T cells, which are the main reservoir of latent infection (241, 242).

The high metabolic state is caused by the constant maintenance of the mucosal immune response against foreign pathogens by Th17 cells, while DCs active Th17 polarization after being exposed to complement opsonized HIV (243). The complement system has been the subject of a few studies. It was soon shown that HIV exhibits on the lipid membrane complement inhibitory molecules CD55 and CD59, which protect the virus from complement virolysis (244, 245). However, activation of the complement system from host-HIV interaction is mainly investigated as it makes HIV more accessible to host cells (246). In fact, complement opsonization of HIV results in higher infectivity and viral transfer from DCs to T cells in a CR3 and DC-SIGN-dependent matter (247), but also transfer from Langerhans cells (248) and causes a higher expression of genes and proteins involved in viral replication and other aspects of the infection (249). Moreover, opsonization reduces antiviral and inflammatory responses compared to free HIV (250, 251). A recent study has examined the effects of both free and opsonized HIV on the initial response in colorectal mucosa. Free HIV leads to a strong initial (at 24h) antiviral response (type I IFNs) creating an environment with a lower level of infection than opsonized HIV. Opsonization, instead, first suppresses the immune response, allowing for higher HIV-infected CD4^+^ T cells, DCs, and macrophages at a later time (at 96h). Opsonization alters the activation of signaling pathways, it lowers the level of CD8^+^ T cells expressing perforin and reduces the levels of colorectal CD8^+^ T cells expressing PD-1, a protein that regulates the immune response. Overall, opsonization creates an environment that stimulates mucosal T cell activation and inflammatory T helper cells promoting viral establishment (246).

### 5.1 Anorectal microbiota

The involvement of the rectal microbiota during HIV infection has been explored in recent animal and human studies considering the association with the immune system. Anal microbiota could regulate local inflammation during receptive anal intercourse driving mucosal immune response (252). The most abundant phyla presented in the rectal microbiota are: *Firmicutes, Bacteroidetes, Proteobacteria, Actinobacteria*, and *Fusobacteria*, ranked in order of abundance (252). Studies of the rectal/anal microbiota in HIV-infected and non-infected patients showed a reduction of microbiota diversity (alpha diversity) resulting in a shift of commensal bacteria composition toward a more pathogenic one. Compositional changes in the rectal microbiota were evident beyond the *Prevotella/Bacteroides* clusters with a shift from *Bacteroides* to *Prevotella* (253). Moreover, a study performed on macaques demonstrated that the cohort of animals more susceptible to SHIV infection had high levels of immune activation linked to lower *Bacteroides* and *Firmicutes*, and higher proportions of *Prevotella* spp. in the anal tract (254). Different studies observed that the rectal mucosa of uninfected-HIV men who have sex with uninfected-HIV men (MSM) have a substantial inflammatory state, including the composition of the microbiota with a high *Prevotella-to-Bacteroides* ratio (233, 255). This finding highlights *Prevotella*’s inflammatory role and suggests that this bacterium may contribute to HIV acquisition (**Figure 3**).

The presence of HIV infection disrupted the local mucosal immunity and modified the relative abundance of several genera, including *Gardnerella, Lactobacillus, Corynebacterium*, and *Sutterella*, leading to microbial dysbiosis and possible opportunistic infections (256). Chlamydial and gonococcal rectal infections are the most common sexually transmitted rectal infections among MSM caused by *Chlamydia trachomatis* and *Neisseria gonorrhoeae*. These infections are associated with an increased risk of HIV infection transmission (257). As described for the previous mucosae, the anorectal mucosa also exhibits a correlation between HIV infections and opportunistic infections, which further supports HIV mucosal transmission (256, 257). Moreover, HIV infection facilitates the persistence of mucosal HPV and increases both the risk of anal squamous intraepithelial neoplasia (AIN) and the progression from low-grade (LSIL) to high-grade intraepithelial lesions (HSIL) (257).

## 6 Conclusions

HIV is primarily a sexually transmitted infection, but the overall risk per sexual exposure is low. One of the reasons for this discrepancy lies in the strong protection given by the mucosal innate immune system during the early steps of infection that need to be fully characterized. Understanding these protective mechanisms is crucial not only to better characterize HIV infection, but also because it could help the development of an effective prophylactic or therapeutic therapy. This urgency is underlined by the UNAIDS’ 95-95-95 targets.

In mucosal transmission, differences in histological structures and in the presence of innate immunological components result in a different efficacy of HIV transmission at the mucosal surfaces considered. Impaired mucosal integrity facilitates the infection, while the induction of a pro-inflammatory environment and changes in the composition of the mucosa microbiota promote its establishment. Although some recent advances in understanding the molecular mechanisms of HIV infection and activation of innate immunity, the difficulty in the characterization of the early mucosal responses after HIV infection has led to a paucity of information about the specific events put in place by the cellular innate immune component. The cellular mechanisms adopted by innate immunity assume a broader significance in mucosal protection against HIV transmission as they naturally modulate host early responses against infections, but they also represent a bridge for the induction of adaptive immunity. To our knowledge, mucosal DCs and macrophages are the first sentinels that can capture the virus, but how this sensing leads to CD4^+^ T cell infection and shaping of an adaptive response is still not completely clear. Another unresolved question concerns the escape mechanisms placed by HIV to elude innate sensing. Additional studies should be conducted to better understand the role of MAIT and iNKT during HIV, for example, if these cells are able to prevent acute infection in CD4^+^ T cells (8).

Nevertheless, mucosal vaccination strategies against oral and other mucosal HIV transmissions are under intense research, but the lack of consensus on immune correlates of protection and effective mucosal adjuvants and delivery systems hamper progress toward an effective vaccine. Immunological containment of HIV infection in the mucosa would represent a strategic choice to effectively block viral replication and prevent its peripheral spread and systemic dissemination. Further studies are needed to assess whether a systemic immune response is an appropriate objective, or whether predominantly mucosal or both mucosal and systemic immunity would be more effective (258–261).

The elicitation of specific humoral responses plays a central role in mucosal protection, as demonstrated by a recent study that describes how intravenous administration of a neutralizing monoclonal antibody led to its distribution into the female genital and male rectal mucosa, retaining its anti–HIV-1 functionality (262). However, the viral clearance mediated by innate immunity is a fundamental determinant of vaccine efficacy. In particular, the use of specific adjuvants able to stimulate different innate immune cells represents a possible approach to shape mucosal immune responses against HIV infection (260), underlining once again how the precise understanding of the molecular mechanisms that promote innate immune response is pivotal for the development of effective vaccine formulations. The question remains whether local vaccination is superior to a systemic or distant mucosal vaccine for protection from infection or disease in clinical settings. Based on observations on how HIV and simian immunodeficiency virus (SIV) are transmitted mucosally and spread systemically, a multi-level barrier model that would confer generalized immunity against HIV transmission has been proposed (263, 264).

Vaccination against oral transmission is a new field and requires more in-depth studies. Oral vaccines are attractive because they can induce high intestinal immunity, are relatively non-invasive, and can be administered on a large scale (265). An alternative strategy of oral vaccination is to directly target the tissues within the oral mucosa for antigen delivery, targeting the buccal (inner cheek, B) and sublingual (below the tongue, SL) tissue (266, 267). A recent study demonstrates the vaccine-mediated protection of MVA-HIV/cycP-gp120 immunization against a pathogenic, heterologous SHIV, as well as the viability and effectiveness of needle-free SL/B immunization as an alternative to conventional needle-based vaccination (268). Although oral, sublingual, and nasal routes are more convenient, vaccination in the genital tract could have significant advantages in targeting STDs, even as a vaccine-boosting approach. A vaccine trial in women was described in which a vaccine consisting of HIV-1 gp140 linked to the chaperone 70-kDa heat shock protein (HSP70) was administered by the vaginal mucosal route (269). The results demonstrated that the immunization led to *ex vivo* inhibition of HIV-1 replication and early innate response in women. The rectal route of transmission is important not only among homosexuals, but it is becoming increasingly apparent as an important route of transmission among heterosexuals who engage anal sex. Although there is some preliminary evidence that oral immunization can protect against rectal HIV transmission, general vaccination studies suggest that rectal immunization may prove to be more protective. To date, few *in vivo* studies have compared rectal vs. oral or vaginal vaccination, and protection against rectal challenge, using recombinant HIV-1 Gag p24 protein plus cholera toxin and HIV-DNA, respectively (270, 271).

The most important obstacle to mucosal immunization, including those against HIV, is the design and development of safe and effective immune adjuvants and delivery systems. The use of live attenuated viral and bacterial delivery systems would most likely reduce the need for adjuvants. However, live attenuated delivery systems present safety and anti-vector pre-existing immunity issues. On the other hand, DNA- and protein-based vaccines require the use of adjuvants. There is a current need to find safe and effective formulations, particularly those that can induce a Th1-type immune response, or at least do not cause an overt Th2-type immune response (272). This is because a Th2-inducing adjuvant given intra-nasally could result in adverse mucus production, vasoconstriction, and asthma-like symptoms.

It has been suggested that microbiome modulation through probiotic therapy increases mucosal immunity. Previous studies have confirmed that probiotics are well-tolerated by HIV-infected individuals under ART, although the overall conclusions varied between studies (273). Recent work has theorized that the immunologic shifts induced by probiotic therapy could simultaneously enhance SIV/HIV vaccine-specific mucosal immunity while limiting the accumulation of potential SHIV cells (274). The data indicate that although the SIV/HIV DNA/protein co-immunization strategy elicited both T and B cell adaptive responses *in vivo* in rhesus macaques, it did not protect from the heterologous, intrarectal SHIV.CH505 challenge. However, an exploratory study identified pre-existing gut microbial and immune activation signatures as potential predictors of sustained HIV-1 control in the absence of ART, providing a potential target for future treatment strategies and opening new chances for a functional HIV cure (275).

An alternative therapy strategy could be based on iNKT as a candidate for the generation of innovative anti-HIV chimeric antigen receptor T (CAR-T). These cells have many advantages over conventional T-cells as potent cytotoxicity and an improved safety profile due to the lack of MHC restriction (276–278).

Lastly, findings that some infections induce immunity not only against the causative agent but also against unrelated pathogens, have been proposed in the context of HIV infection. The mechanisms behind this phenomenon have started to be identified only recently. It was found that the key cells responsible for heterologous protection are innate immune cells such as NKs, DC, and monocytes/macrophages. These hyper-responsive cells may be the cause of sustained inflammation, which underlies most comorbidities associated with HIV infection, even if successfully managed by ART. Altered epigenetic profiles such as DNA methylation have been reported in HIV-infected individuals (279–281). Specific profiles have been associated with progressive aging and non-AIDS–related comorbidities, such as insulin resistance, neurocognitive disorders, and chronic kidney disease (282–284). Interestingly, HIV/SIV DNA vaccination was shown to induce a trained immunity phenotype *in vivo* through the upregulation of IL-1β–related genes, which correlated with protection against subsequent SIV infection in macaques (285). At the same time, there is increasing evidence that some infections may increase susceptibility to HIV infection. As demonstrated by Jensen et al. (286), non-specific induction of trained immunity may not be an effective approach for HIV-1 elite control due to possible off-target effects on other immune cells. Instead, infusion of specific innate effector cells exhibiting enhanced immunity, such as CAR expression (knockout of anti-inflammatory genes), and overexpression of innate effector molecules could be a more feasible strategy to utilize trained immunity for HIV-1 immunotherapeutics (287).

Whether there will be a vaccine that will protect against transmission through all the routes that have been discussed here, is the subject of future research endeavors. As of now, almost all investigators focus on a single mucosal route of transmission. The design of a vaccine that can protect any of these mucosal routes against transmission will be a great achievement and will also pave the way for protection against transmission through the other routes.

The study of all the aspects involved in the mucosal HIV transmission is significant to gain information and expand the knowledge that could help vaccine design and adjuvants choice for HIV infection eradication.

## Author contributions

All authors contributed to the article and approved the submitted version.

## References

[1] Frescura L Godfrey-Faussett P A El-Sadr W Syarif O Ghys PD. Group on and behalf of the 2025 testing treatment target w. achieving the 95 95 95 targets for all: A pathway to ending AIDS. PloS One (2022) 17:e0272405. doi: 10.1371/journal.pone.0272405 35925943 PMC9352102

[2] HIV.gov. HIV.gov global statistics (2022). Available at: https://www.hiv.gov/hiv-basics/overview/data-and-trends/global-statistics (Accessed November 15, 2022).

[3] Shi Y Su J Chen R Wei W Yuan Z Chen X. The role of innate immunity in natural elite controllers of HIV-1 infection. Front Immunol (2022) 13:780922. doi: 10.3389/fimmu.2022.780922 35211115 PMC8861487

[4] Saez R Echaniz P Juan MDD Iribarren JA Cuadrado E. The impaired response of NK cells from HIV-infected progressor patients to a-class CpG oligodeoxynucleotides is largely dependent of a decreased production of IL-12. Immunol Lett (2007) 109:83–90. doi: 10.1016/j.imlet.2007.01.006 17343921

[5] Herbeuval J-P Nilsson J Boasso A Hardy AW Kruhlak MJ Anderson SA. Differential expression of IFN-α and TRAIL/DR5 in lymphoid tissue of progressor versus nonprogressor HIV-1-infected patients. Proc Natl Acad Sci (2006) 103:7000–5. doi: 10.1073/pnas.0600363103 PMC1444883 16632604

[6] HacksteinC-PKlenermanP. Emerging features of MAIT cells and other unconventional T cell populations in human viral disease and vaccination. Semin Immunol (2022) 61:101661. doi: 10.1016/j.smim.2022.101661 36374780

[7] Sandberg JK Fast NM Palacios EH Fennelly G Dobroszycki J Palumbo P. Selective loss of innate CD4 + Vα24 natural killer T cells in human immunodeficiency virus infection. J Virol (2002) 76:7528–34. doi: 10.1128/jvi.76.15.7528-7534.2002 PMC136353 12097565

[8] Juno JA Phetsouphanh C Klenerman P Kent SJ. Perturbation of mucosal-associated invariant T cells and iNKT cells in HIV infection. Curr Opin HIV AIDS (2019) 14:77–84. doi: 10.1097/coh.0000000000000526 30585802

[9] Su B Kong D Yang X Zhang T Kuang Y. Mucosal-associated invariant T cells: A cryptic coordinator in HIV-infected immune reconstitution. J Med Virol (2022) 94:3043–53. doi: 10.1002/jmv.27696 35243649

[10] Vitinghoff E Douglas J Judon F McKiman D MacQueen K Buchinder SP. Per-contact risk of human immunodificiency virus tramnsmision between Male sexual partners. Am J Epidemiol (1999) 150:306–11. doi: 10.1093/oxfordjournals.aje.a010003 10430236

[11] Leynaert B Downs AM Vincenzi I. Heterosexual transmission of human immunodeficiency VirusVariability of infectivity throughout the course of infection. Am J Epidemiol (1998) 148:88–96. doi: 10.1093/oxfordjournals.aje.a009564 9663408

[12] Jin F Jansson J Law M Prestage GP Zablotska I Imrie JC. Per-contact probability of HIV transmission in homosexual men in Sydney in the era of HAART. Aids (2010) 24:907–13. doi: 10.1097/qad.0b013e3283372d90 PMC2852627 20139750

[13] DeGruttola V Seage GR Mayer KH Horsburgh CR. Infectiousness of HIV between male homosexual partners. J Clin Epidemiol (1989) 42:849–56. doi: 10.1016/0895-4356(89)90098-x 2789269

[14] Boily M-C Baggaley RF Wang L Masse B White RG Hayes RJ. Heterosexual risk of HIV-1 infection per sexual act: systematic review and meta-analysis of observational studies. Lancet Infect Dis (2009) 9:118–29. doi: 10.1016/s1473-3099(09)70021-0 PMC4467783 19179227

[15] Romero Jd Marincovich B Castilla J García S Campo J Hernando V. Evaluating the risk of HIV transmission through unprotected orogenital sex. Aids (2002) 16:1296–7. doi: 10.1097/00002030-200206140-00017 12045500

[16] Patel P Borkowf CB Brooks JT Lasry A Lansky A Mermin J. Estimating per-act HIV transmission risk. Aids (2014) 28:1509–19. doi: 10.1097/qad.0000000000000298 PMC6195215 24809629

[17] Prevention c for DC and. CDC HIV basic statistics (2022). Available at: https://www.cdc.gov/hiv/basics/statistics.html (Accessed November 15, 2022).

[18] Cohen T Cohen SJ Antonovsky N Cohen IR Shai Y. HIV-1 gp41 and TCRα trans-membrane domains share a motif exploited by the HIV virus to modulate T-cell proliferation. PloS Pathog (2010) 6:e1001085. doi: 10.1371/journal.ppat.1001085 20824090 PMC2932719

[19] Blood’ GACB (Arbeitskreis B) Subgroup ‘Assessment of Pathogens Transmissible. Human immunodeficiency virus (HIV). Transfus Med Hemoth (2016) 43:203–22. doi: 10.1159/000445852 PMC4924471 27403093

[20] Arrildt KT Joseph SB Swanstrom R. The HIV-1 env protein: A coat of many colors. Curr Hiv-aids Rep (2012) 9:52–63. doi: 10.1007/s11904-011-0107-3 22237899 PMC3658113

[21] Neidleman JA Chen JC Kohgadai N Müller JA Laustsen A Thavachelvam K. Mucosal stromal fibroblasts markedly enhance HIV infection of CD4+ T cells. PloS Pathog (2017) 13:e1006163. doi: 10.1371/journal.ppat.1006163 28207890 PMC5312882

[22] Dorosko SM Connor RI. Primary human mammary epithelial cells endocytose HIV-1 and facilitate viral infection of CD4+ T lymphocytes. J Virol (2010) 84:10533–42. doi: 10.1128/jvi.01263-10 PMC2950585 20702626

[23] Gonzalez SM Aguilar-Jimenez W Su R-C Rugeles MT. Mucosa: Key interactions determining sexual transmission of the HIV infection. Front Immunol (2019) 10:144. doi: 10.3389/fimmu.2019.00144 30787929 PMC6373783

[24] Yin X Langer S Zhang Z Herbert KM Yoh S König R. Sensor sensibility–HIV-1 and the innate immune response. Cells (2020) 9:254. doi: 10.3390/cells9010254 31968566 PMC7016969

[25] Altfeld M Gale M Jr. Innate immunity against HIV-1 infection. Nat Immunol (2015) 16:554–62. doi: 10.1038/ni.3157 25988887

[26] Sego TJ Aponte-Serrano JO Gianlupi JF Heaps SR Breithaupt K Brusch L. A modular framework for multiscale, multicellular, spatiotemporal modeling of acute primary viral infection and immune response in epithelial tissues and its application to drug therapy timing and effectiveness. PloS Comput Biol (2020) 16:e1008451. doi: 10.1371/journal.pcbi.1008451 33347439 PMC7785254

[27] Sperk M Ambikan AT Ray S Singh K Mikaeloff F Diez RC. Fecal metabolome signature in the HIV-1 elite control phenotype: Enrichment of dipeptides acts as an HIV-1 antagonist but a prevotella agonist. J Virol (2021) 95:e00479–21. doi: 10.1128/jvi.00479-21 PMC8387056 34232744

[28] Tugizov S. Human immunodeficiency virus-associated disruption of mucosal barriers and its role in HIV transmission and pathogenesis of HIV/AIDS disease. Tissue Barriers (2016) 4:e1159276. doi: 10.1080/21688370.2016.1159276 27583187 PMC4993574

[29] Groeger S Meyle J. Oral mucosal epithelial cells. Front Immunol (2019) 10:208. doi: 10.3389/fimmu.2019.00208 30837987 PMC6383680

[30] Pelaez-Prestel HF Sanchez-Trincado JL Lafuente EM Reche PA. Immune tolerance in the oral mucosa. Int J Mol Sci (2021) 22:12149. doi: 10.3390/ijms222212149 34830032 PMC8624028

[31] Squier CA Kremer MJ. Biology of oral mucosa and esophagus. Jnci Monogr (2001) 2001:7–15. doi: 10.1093/oxfordjournals.jncimonographs.a003443 11694559

[32] Tugizov SM Herrera R Veluppillai P Greenspan D Soros V Greene WC. Differential transmission of HIV traversing fetal Oral/Intestinal epithelia and adult oral epithelia. J Virol (2012) 86:2556–70. doi: 10.1128/jvi.06578-11 PMC3302289 22205732

[33] Shattock RJ Moore JP. Inhibiting sexual transmission of HIV-1 infection. Nat Rev Microbiol (2003) 1:25–34. doi: 10.1038/nrmicro729 15040177

[34] Reich O Regauer S McCluggage WG Bergeron C Redman C. Defining the cervical transformation zone and squamocolumnar junction. Int J Gynecol Pathol (2017) 36:517–22. doi: 10.1097/pgp.0000000000000381 28639968

[35] Pudney J Quayle AJ Anderson DJ. Immunological microenvironments in the human vagina and cervix: Mediators of cellular immunity are concentrated in the cervical transformation zone. Biol Reprod (2005) 73:1253–63. doi: 10.1095/biolreprod.105.043133 16093359

[36] Taves DR. The intromission function of the foreskin. Med Hypotheses (2002) 59:180–2. doi: 10.1016/s0306-9877(02)00250-5 12208206

[37] Russo CL Spurr-Michaud S Tisdale A Pudney J Anderson D Gipson IK. Mucin gene expression in human male urogenital tract epithelia. Hum Reprod (2006) 21:2783–93. doi: 10.1093/humrep/del164 PMC2893033 16997931

[38] Haase AT. Targeting early infection to prevent HIV-1 mucosal transmission. Nature (2010) 464:217–23. doi: 10.1038/nature08757 20220840

[39] Lyons KM Butler SL. Anal intraepithelial neoplasia from a pathologists point of view. Clin Colon Rect Surg (2018) 31:328–35. doi: 10.1055/s-0038-1668102 PMC6214808 30397392

[40] Padian NS Straten Avd Ramjee G Chipato T Bruyn G Blanchard K. Diaphragm and lubricant gel for prevention of HIV acquisition in southern African women: a randomised controlled trial. Lancet (2007) 370:251–61. doi: 10.1016/s0140-6736(07)60950-7 PMC2442038 17631387

[41] Scully C Porter S. The level of risk of transmission of human immunodeficiency virus between patients and dental staff. Brit Dent J (1991) 170:97–100. doi: 10.1038/sj.bdj.4807440 2007078

[42] Kordy K Tobin NH Aldrovandi GM. HIV And SIV in body fluids: From breast milk to the genitourinary tract. Curr Immunol Rev (2019) 15:139–52. doi: 10.2174/1573395514666180605085313 PMC7730163 33312088

[43] Joag SV Adany I Li Z Foresman L Pinson DM Wang C. Animal model of mucosally transmitted human immunodeficiency virus type 1 disease: intravaginal and oral deposition of simian/human immunodeficiency virus in macaques results in systemic infection, elimination of CD4+ T cells, and AIDS. J Virol (1997) 71:4016–23. doi: 10.1128/jvi.71.5.4016-4023.1997 PMC191554 9094679

[44] Liu X Zha J Chen H Nishitani J Camargo P Cole SW. Human immunodeficiency virus type 1 infection and replication in normal human oral keratinocytes. J Virol (2003) 77:3470–6. doi: 10.1128/jvi.77.6.3470-3476.2003 PMC149546 12610122

[45] Vacharaksa A Asrani AC Gebhard KH Fasching CE Giacaman RA Janoff EN. Oral keratinocytes support non-replicative infection and transfer of harbored HIV-1 to permissive cells. Retrovirology (2008) 5:66. doi: 10.1186/1742-4690-5-66 18637194 PMC2491655

[46] Kohli A Islam A Moyes DL Murciano C Shen C Challacombe SJ. Oral and vaginal epithelial cell lines bind and transfer cell-free infectious HIV-1 to permissive cells but are not productively infected. PloS One (2014) 9:e98077. doi: 10.1371/journal.pone.0098077 24857971 PMC4032250

[47] Moyes D Islam A Kohli A Naglik J. Oral epithelial cells and their interactions with HIV-1. Oral Dis (2016) 22:66–72. doi: 10.1111/odi.12410 26879550

[48] Geijtenbeek TBH Kwon DS Torensma R Vliet SJv Duijnhoven GCFv Middel J. DC-SIGN, a dendritic cell–specific HIV-1-Binding protein that enhances trans-infection of T cells. Cell (2000) 100:587–97. doi: 10.1016/s0092-8674(00)80694-7 10721995

[49] Harouse JM Bhat S Spitalnik SL Laughlin M Stefano K Silberberg DH. Inhibition of entry of HIV-1 in neural cell lines by antibodies against galactosyl ceramide. Science (1991) 253:320–3. doi: 10.1126/science.1857969 1857969

[50] Alfsen A Bomsel M. HIV-1 gp41 envelope residues 650–685 exposed on native virus act as a lectin to bind epithelial cell galactosyl ceramide*. J Biol Chem (2002) 277:25649–59. doi: 10.1074/jbc.m200554200 11940580

[51] Bobardt MD Saphire ACS Hung H-C Yu X Schueren BVd Zhang Z. Syndecan captures, protects, and transmits HIV to T lymphocytes. Immunity (2003) 18:27–39. doi: 10.1016/s1074-7613(02)00504-6 12530973

[52] Kohli A Islam A Moyes DL Murciano C Shen C Challacombe SJ. Correction: Oral and vaginal epithelial cell lines bind and transfer cell-free infectious HIV-1 to permissive cells but are not productively infected. PloS One (2020) 15:e0229553. doi: 10.1371/journal.pone.0229553 32074132 PMC7029853

[53] Izquierdo-Useros N Lorizate M McLaren PJ Telenti A Kräusslich H-G Martinez-Picado J. HIV-1 capture and transmission by dendritic cells: The role of viral glycolipids and the cellular receptor siglec-1. PloS Pathog (2014) 10:e1004146. doi: 10.1371/journal.ppat.1004146 25033082 PMC4102576

[54] Challacombe S Sweet S. Oral mucosal immunity and HIV infection: current status. Oral Dis (2002) 8:55–62. doi: 10.1034/j.1601-0825.2002.00013.x 12164661

[55] Hussain LA Lehner T. Comparative investigation of langerhans’ cells and potential receptors for HIV in oral, genitourinary and rectal epithelia. Immunology (1995) 85:475–84. PMC1383923 7558138

[56] Sufiawati I Tugizov SM. HIV-Associated disruption of tight and adherens junctions of oral epithelial cells facilitates HSV-1 infection and spread. PloS One (2014) 9:e88803–14. doi: 10.1371/journal.pone.0088803 PMC3931628 24586397

[57] Sufiawati I Tugizov SM. HIV-Induced matrix metalloproteinase-9 activation through mitogen-activated protein kinase signalling promotes HSV-1 cell-to-cell spread in oral epithelial cells. J Gen Virol (2018) 99:937–47. doi: 10.1099/jgv.0.001075 PMC6537617 29775175

[58] Tugizov SM Herrera R Veluppillai P Greenspan D Soros V Greene WC. HIV Is inactivated after transepithelial migration *via* adult oral epithelial cells but not fetal epithelial cells. Virology (2011) 409:211–22. doi: 10.1016/j.virol.2010.10.004 PMC3034249 21056450

[59] Bomsel M. Transcytosis of infectious human immunodeficiency virus across a tight human epithelial cell line barrier. Nat Med (1997) 3:42–7. doi: 10.1038/nm0197-42 8986739

[60] Bobardt MD Chatterji U Selvarajah S Schueren BVd David G Kahn B. Cell-free human immunodeficiency virus type 1 transcytosis through primary genital epithelial cells. J Virol (2007) 81:395–405. doi: 10.1128/jvi.01303-06 17050597 PMC1797244

[61] Kazmi SH Naglik JR Sweet SP Evans RW O’Shea S Banatvala JE. Comparison of human immunodeficiency virus type 1-specific inhibitory activities in saliva and other human mucosal fluids. Clin Vaccine Immunol (2006) 13:1111–8. doi: 10.1128/cdli.00426-05 PMC1595323 16928883

[62] Lü FX Jacobson RS. Oral mucosal immunity and HIV/SIV infection. J Dent Res (2007) 86:216–26. doi: 10.1177/154405910708600305 17314252

[63] Baron S Poast J Cloyd MW. Why is HIV rarely transmitted by oral secretions?: saliva can disrupt orally shed, infected leukocytes. Arch Intern Med (1999) 159:303–10. doi: 10.1001/archinte.159.3.303 9989543

[64] Baron S Poast J Richardson CJ Nguyen D Cloyd M. Oral transmission of human immunodeficiency virus by infected seminal fluid and milk: A novel mechanism. J Infect Dis (2000) 181:498–504. doi: 10.1086/315251 10669332

[65] Bergey EJ Cho MI Blumberg BM Hammarskjöld ML Rekosh D Epstein LG. Interaction of HIV-1 and human salivary mucins. J Acq Immun Def Synd (1994) 7:995–1002. 8083829

[66] Nagashunmugam T Malamud D Davis C Abrams WR Friedman HM. Human submandibular saliva inhibits human immunodeficiency virus type 1 infection by displacing envelope glycoprotein gp120 from the virus. J Infect Dis (1998) 178:1635–41. doi: 10.1086/314511 9815215

[67] Crombie R Silverstein RL MacLow C Pearce SFA Nachman RL Laurence J. Identification of a CD36-related thrombospondin 1–binding domain in HIV-1 envelope glycoprotein gp120: Relationship to HIV-1–specific inhibitory factors in human saliva. J Exp Med (1998) 187:25–35. doi: 10.1084/jem.187.1.25 9419208 PMC2199189

[68] Robinovitch MR Ashley RL Iversen JM Vigoren EM Oppenheim FG Lamkin M. Parotid salivary basic proline-rich proteins inhibit HIV-I infectivity. Oral Dis (2001) 7:86–93. doi: 10.1034/j.1601-0825.2001.70204.x 11355444

[69] Swart PJ Kuipers EM Smit C Strate BWVD Harmsen MC Meijer DK. Lactoferrin. antiviral activity of lactoferrin. Adv Exp Med Biol (1998) 443:205–13. doi: 10.1007/978-1-4757-9068-9_24 9781360

[70] Kell DB Heyden EL Pretorius E. The biology of lactoferrin, an iron-binding protein that can help defend against viruses and bacteria. Front Immunol (2020) 11:1221. doi: 10.3389/fimmu.2020.01221 32574271 PMC7271924

[71] Quiñones-Mateu ME Lederman MM Feng Z Chakraborty B Weber J Rangel HR. Human epithelial β-defensins 2 and 3 inhibit HIV-1 replication. Aids (2003) 17:F39–48. doi: 10.1097/00002030-200311070-00001 14571200

[72] Lafferty MK Sun L DeMasi L Lu W Garzino-Demo A. CCR6 ligands inhibit HIV by inducing APOBEC3G. Blood (2010) 115:1564–71. doi: 10.1182/blood-2009-06-226423 PMC2830761 20023216

[73] Lafferty MK Sun L Christensen-Quick A Lu W Garzino-Demo A. Human beta defensin 2 selectively inhibits HIV-1 in highly permissive CCR6+CD4+ T cells. Viruses (2017) 9:111. doi: 10.3390/v9050111 28509877 PMC5454423

[74] Feng Z Dubyak GR Lederman MM Weinberg A. Cutting edge: Human β defensin 3–a novel antagonist of the HIV-1 coreceptor CXCR4. J Immunol (2006) 177:782–6. doi: 10.4049/jimmunol.177.2.782 16818731

[75] Herrera R Morris M Rosbe K Feng Z Weinberg A Tugizov S. Human beta-defensins 2 and -3 cointernalize with human immunodeficiency virus *via* heparan sulfate proteoglycans and reduce infectivity of intracellular virions in tonsil epithelial cells. Virology (2016) 487:172–87. doi: 10.1016/j.virol.2015.09.025 PMC4679645 26539799

[76] Wang G Watson KM Buckheit RW. Anti-human immunodeficiency virus type 1 activities of antimicrobial peptides derived from human and bovine cathelicidins. Antimicrob Agents Ch (2008) 52:3438–40. doi: 10.1128/aac.00452-08 PMC2533476 18591279

[77] Steinstraesser L Tippler B Mertens J Lamme E Homann H-H Lehnhardt M. Inhibition of early steps in the lentiviral replication cycle by cathelicidin host defense peptides. Retrovirology (2005) 2:2. doi: 10.1186/1742-4690-2-2 15656908 PMC548510

[78] Bergman P Walter-Jallow L Broliden K Agerberth B Soderlund J. The antimicrobial peptide LL-37 inhibits HIV-1 replication. Curr HIV Res (2007) 5:410–5. doi: 10.2174/157016207781023947 17627504

[79] Wong JH Legowska A Rolka K Ng TB Hui M Cho CH. Effects of cathelicidin and its fragments on three key enzymes of HIV-1. Peptides (2011) 32:1117–22. doi: 10.1016/j.peptides.2011.04.017 21539873

[80] Shugars DC. Endogenous mucosal antiviral factors of the oral cavity. J Infect Dis (1999) 179:S431–5. doi: 10.1086/314799 10099113

[81] McNeely TB Dealy M Dripps DJ Orenstein JM Eisenberg SP Wahl SM. Secretory leukocyte protease inhibitor: a human saliva protein exhibiting anti-human immunodeficiency virus 1 activity *in vitro* . J Clin Invest (1995) 96:456–64. doi: 10.1172/jci118056 PMC185219 7615818

[82] McNeely TB Shugars DC Rosendahl M Tucker C Eisenberg SP Wahl SM. Inhibition of human immunodeficiency virus type 1 infectivity by secretory leukocyte protease inhibitor occurs prior to viral reverse transcription. Blood (1997) 90:1141–9. doi: 10.1182/blood.V90.3.1141 9242546

[83] Farquhar C VanCott TC Mbori-Ngacha DA Horani L Bosire RK Kreiss JK. Salivary secretory leukocyte protease inhibitor is associated with reduced transmission of human immunodeficiency virus type 1 through breast milk. J Infect Dis (2002) 186:1173–6. doi: 10.1086/343805 PMC3382060 12355371

[84] Becquart P Grésenguet G Hocini H Kazatchkine MD Bélec L. Secretory leukocyte protease inhibitor in colostrum and breast milk is not a major determinant of the protection of early postnatal transmission of HIV. Aids (1999) 13:2599. doi: 10.1097/00002030-199912240-00018 10630534

[85] Nittayananta W Amornthatree K Kemapunmanus M Talungchit S Sriplung H. Expression of oral cytokines in HIV-infected subjects with long-term use of antiretroviral therapy. Oral Dis (2014) 20:e57–64. doi: 10.1111/odi.12135 PMC3859523 23718561

[86] Li H Reeves RK. Functional perturbation of classical natural killer and innate lymphoid cells in the oral mucosa during SIV infection. Front Immunol (2013) 3:417. doi: 10.3389/fimmu.2012.00417 23316201 PMC3539714

[87] George MD Verhoeven D Sankaran S Glavan T Reay E Dandekar S. Heightened cytotoxic responses and impaired biogenesis contribute to early pathogenesis in the oral mucosa of simian immunodeficiency virus-infected rhesus macaques. Clin Vaccine Immunol (2009) 16:277–81. doi: 10.1128/cvi.00265-08 PMC2643550 19091994

[88] Santo JPD Vosshenrich CA Satoh-Takayama N. A ‘natural’ way to provide innate mucosal immunity. Curr Opin Immunol (2010) 22:435–41. doi: 10.1016/j.coi.2010.05.004 20573491

[89] Wills-Karp M Finkelman FD. Innate lymphoid cells wield a double-edged sword. Nat Immunol (2011) 12:1025–7. doi: 10.1038/ni.2142 22012433

[90] Reeves RK Rajakumar PA Evans TI Connole M Gillis J Wong FE. Gut inflammation and indoleamine deoxygenase inhibit IL-17 production and promote cytotoxic potential in NKp44+ mucosal NK cells during SIV infection. Blood (2011) 118:3321–30. doi: 10.1182/blood-2011-04-347260 PMC3179400 21791421

[91] Rosero EP Heron S Jovel J O’Neil CR Turvey SL Parashar P. Differential signature of the microbiome and neutrophils in the oral cavity of HIV-infected individuals. Front Immunol (2021) 12:780910. doi: 10.3389/fimmu.2021.780910 34858437 PMC8630784

[92] Casulli S Elbim C. Interactions between human immunodeficiency virus type 1 and polymorphonuclear neutrophils. J Innate Immun (2014) 6:13–20. doi: 10.1159/000353588 23867213 PMC6741617

[93] Samaranayake L Matsubara VH. Normal oral flora and the oral ecosystem. Dent Clin N Am (2017) 61:199–215. doi: 10.1016/j.cden.2016.11.002 28317562

[94] Li J Chang S Guo H Ji Y Jiang H Ruan L. Altered salivary microbiome in the early stage of HIV infections among young Chinese men who have sex with men (MSM). Pathogens (2020) 9:960. doi: 10.3390/pathogens9110960 33228000 PMC7699166

[95] Yang L Dunlap DG Qin S Fitch A Li K Koch CD. Alterations in oral microbiota in HIV are related to decreased pulmonary function. Am J Resp Crit Care (2019) 201:445–57. doi: 10.1164/rccm.201905-1016oc PMC7049920 31682463

[96] Mukherjee PK Chandra J Retuerto M Tatsuoka C Ghannoum MA McComsey GA. Dysbiosis in the oral bacterial and fungal microbiome of HIV-infected subjects is associated with clinical and immunologic variables of HIV infection. PloS One (2018) 13:e0200285. doi: 10.1371/journal.pone.0200285 29995962 PMC6040710

[97] Kistler JO Arirachakaran P Poovorawan Y Dahlén G Wade WG. The oral microbiome in human immunodeficiency virus (HIV)-positive individuals. J Med Microbiol (2015) 64:1094–101. doi: 10.1099/jmm.0.000128 26297584

[98] Moyes D Saxena D John M Malamud D. The gut and oral microbiome in HIV disease: a workshop report. Oral Dis (2016) 22:166–70. doi: 10.1111/odi.12415 27109284

[99] Saxena D Li Y Yang L Pei Z Poles M Abrams WR. Human microbiome and HIV/AIDS. Curr Hiv-aids Rep (2012) 9:44–51. doi: 10.1007/s11904-011-0103-7 22193889 PMC4154628

[100] Coker MO Mongodin EF El-Kamary SS Akhigbe P Obuekwe O Omoigberale A. Immune status, and not HIV infection or exposure, drives the development of the oral microbiota. Sci Rep-uk (2020) 10:10830. doi: 10.1038/s41598-020-67487-4 PMC7331591 32616727

[101] Hager CL Ghannoum MA. The mycobiome in HIV. Curr Opin HIV AIDS (2018) 13:69–72. doi: 10.1097/coh.0000000000000432 29028668 PMC5805152

[102] Mukherjee PK Chandra J Retuerto M Sikaroodi M Brown RE Jurevic R. Oral mycobiome analysis of HIV-infected patients: Identification of pichia as an antagonist of opportunistic fungi. PloS Pathog (2014) 10:e1003996. doi: 10.1371/journal.ppat.1003996 24626467 PMC3953492

[103] Sardi JCO Duque C Höfling JF Gonçalves RB. Genetic and phenotypic evaluation of candida albicans strains isolated from subgingival biofilm of diabetic patients with chronic periodontitis. Med Mycol (2012) 50:467–75. doi: 10.3109/13693786.2011.633233 22114891

[104] Li S Su B He Q-S Wu H Zhang T. Alterations in the oral microbiome in HIV infection: causes, effects and potential interventions. Chin Med J-peking (2021) 134:2788–98. doi: 10.1097/cm9.0000000000001825 PMC8667981 34670249

[105] Heron SE Elahi S. HIV Infection and compromised mucosal immunity: Oral manifestations and systemic inflammation. Front Immunol (2017) 8:241. doi: 10.3389/fimmu.2017.00241 28326084 PMC5339276

[106] Howati AE Tappuni A. Systematic review of the changing pattern of the oral manifestations of HIV. J Invest Clin Dent (2018) 9:e12351. doi: 10.1111/jicd.12351 30019446

[107] Pour AH Salari S Almani PGN. Oropharyngeal candidiasis in HIV/AIDS patients and HIVfree subjects in the southeast of Iran. Curr Med Mycol (2018) 4:1–6. doi: 10.18502/cmm.4.4.379 PMC6386505 30815610

[108] Khedri S Santos ALS Roudbary M Hadighi R Falahati M Farahyar S. Iranian HIV/AIDS patients with oropharyngeal candidiasis: identification, prevalence and antifungal susceptibility of candida species. Lett Appl Microbiol (2018) 67:392–9. doi: 10.1111/lam.13052 30019443

[109] Kwamin F Nartey NO Codjoe FS Newman MJ. Distribution of candida species among HIV-positive patients with oropharyngeal candidiasis in Accra, Ghana. J Infect Dev Ctries (2013) 7:041–5. doi: 10.3855/jidc.2442 23324819

[110] Gonçalves L de S Ferreira SMS Souza CO Souto R Colombo AP. Clinical and microbiological profiles of human immunodeficiency virus (HIV)–seropositive brazilians undergoing highly active antiretroviral therapy and HIV-seronegative brazilians with chronic periodontitis. J Periodontol (2007) 78:87–96. doi: 10.1902/jop.2007.060040 17199544

[111] Gruffaz M Zhang T Marshall V Gonçalves P Ramaswami R Labo N. Signatures of oral microbiome in HIV-infected individuals with oral kaposi’s sarcoma and cell-associated KSHV DNA. PloS Pathog (2020) 16:e1008114. doi: 10.1371/journal.ppat.1008114 31951641 PMC6992226

[112] Gershon AA. Prevention and treatment of VZV infections in patients with HIV. Herpes J Ihmf (2001) 8:32–6. 11867015

[113] Macphail LA Hilton JF Heinic GS Greenspan D. Direct immunofluorescence vs. culture for detecting hsv in oral ulcers: a comparison. J Am Dent Assoc (1995) 126:74–8. doi: 10.14219/jada.archive.1995.0026 7822648

[114] Regezi JA Eversole LR Barker BF Rick GM Silverman S. Herpes simplex and cytomegalovirus coinfected oral ulcers in HIV-positive patients. Oral Surg Oral Med Oral Pathol Oral Radiol Endodontology (1996) 81:55–62. doi: 10.1016/s1079-2104(96)80149-1 8850485

[115] Syrjänen S Leimola-Virtanen R Schmidt-Westhausen A Reichart PA. Oral ulcers in AIDS patients frequently associated with cytomegalovirus (CMV) and Epstein-Barr virus (EBV) infections. J Oral Pathol Med (1999) 28:204–9. doi: 10.1111/j.1600-0714.1999.tb02025.x 10226942

[116] Ensoli B Lusso P Schachter F Josephs SF Rappaport J Negro F. Human herpes virus-6 increases HIV-1 expression in co-infected T cells *via* nuclear factors binding to the HIV-1 enhancer. EMBO J (1989) 8:3019–27. doi: 10.1002/j.1460-2075.1989.tb08452.x PMC401379 2573513

[117] Lusso P Garzino-Demo A Crowley RW Malnati MS. Infection of gamma/delta T lymphocytes by human herpesvirus 6: transcriptional induction of CD4 and susceptibility to HIV infection. J Exp Med (1995) 181:1303–10. doi: 10.1084/jem.181.4.1303 PMC2191959 7699322

[118] Pauk J Huang M-L Brodie SJ Wald A Koelle DM Schacker T. Mucosal shedding of human herpesvirus 8 in men. New Engl J Med (2000) 343:1369–77. doi: 10.1056/nejm200011093431904 11070101

[119] Blázquez MV Madueño JA Jurado R Fernandez-Areas N Munoz E. Human herpesvirus-6 and the course of human immunodeficiency virus infection. J Acquir Immune Defic Syndromes Hum Retrovirology (1995) 9:389–94. doi: 10.1097/00042560-199508000-00009 7600106

[120] Scully C Laskaris G Pindborg J Porter SR Reichart P. Oral manifestations of HIV infection and their management. i. more common lesions. Oral Surg Oral Med Oral Pathol (1991) 71:158–66. doi: 10.1016/0030-4220(91)90459-p 2003011

[121] Lucht E Biberfeld P Linde A. Epstein-Barr Virus (EBV) DNA in saliva and EBV serology of HIV-1-infected persons with and without hairy leukoplakia. J Infection (1995) 31:189–94. doi: 10.1016/s0163-4453(95)80025-5 8586837

[122] Hickey DK Patel MV Fahey JV Wira CR. Innate and adaptive immunity at mucosal surfaces of the female reproductive tract: stratification and integration of immune protection against the transmission of sexually transmitted infections. J Reprod Immunol (2011) 88:185–94. doi: 10.1016/j.jri.2011.01.005 PMC3094911 21353708

[123] Grant KS Wira CR. Effect of mouse uterine stromal cells on epithelial cell transepithelial resistance (TER) and TNF and TGF release in Culture1. Biol Reprod (2003) 69:1091–8. doi: 10.1095/biolreprod.103.015495 12773432

[124] Kaushic C Ferreira VH Kafka JK Nazli A. REVIEW ARTICLE: HIV infection in the female genital tract: Discrete influence of the local mucosal microenvironment. Am J Reprod Immunol (2010) 63:566–75. doi: 10.1111/j.1600-0897.2010.00843.x 20384619

[125] Schaefer TM Desouza K Fahey JV Beagley KW Wira CR. Toll-like receptor (TLR) expression and TLR-mediated cytokine/chemokine production by human uterine epithelial cells. Immunology (2004) 112:428–36. doi: 10.1111/j.1365-2567.2004.01898.x PMC1782499 15196211

[126] Benjelloun F Quillay H Cannou C Marlin R Madec Y Fernandez H. Activation of toll-like receptors differentially modulates inflammation in the human reproductive tract: Preliminary findings. Front Immunol (2020) 11:1655. doi: 10.3389/fimmu.2020.01655 32849571 PMC7417306

[127] Hart KM Murphy AJ Barrett KT Wira CR Guyre PM Pioli PA. Functional expression of pattern recognition receptors in tissues of the human female reproductive tract. J Reprod Immunol (2009) 80:33–40. doi: 10.1016/j.jri.2008.12.004 19406482 PMC2744441

[128] Machado JR Silva MVd Cavellani CL Reis MAd Monteiro MLG dos R Teixeira V de PA. Mucosal immunity in the female genital tract, HIV/AIDS. BioMed Res Int (2014) 2014:350195. doi: 10.1155/2014/350195 25313360 PMC4181941

[129] Kinlock BL Wang Y Turner TM Wang C Liu B. Transcytosis of HIV-1 through vaginal epithelial cells is dependent on trafficking to the endocytic recycling pathway. PloS One (2014) 9:e96760. doi: 10.1371/journal.pone.0096760 24830293 PMC4022679

[130] Carias AM McCoombe S McRaven M Anderson M Galloway N Vandergrift N. Defining the interaction of HIV-1 with the mucosal barriers of the female reproductive tract. J Virol (2013) 87:11388–400. doi: 10.1128/jvi.01377-13 PMC3807311 23966398

[131] Nazli A Chan O Dobson-Belaire WN Ouellet M Tremblay MJ Gray-Owen SD. Exposure to HIV-1 directly impairs mucosal epithelial barrier integrity allowing microbial translocation. PloS Pathog (2010) 6:e1000852. doi: 10.1371/journal.ppat.1000852 20386714 PMC2851733

[132] Smith SM Mefford M Sodora D Klase Z Singh M Alexander N. Topical estrogen protects against SIV vaginal transmission without evidence of systemic effect. Aids (2004) 18:1637–43. doi: 10.1097/01.aids.0000131393.76221.cc 15280774

[133] Wira CR Fahey JV. A new strategy to understand how HIV infects women&colon; identification of a window of vulnerability during the menstrual cycle. Aids (2008) 22:1909–17. doi: 10.1097/qad.0b013e3283060ea4 PMC2647143 18784454

[134] Selhorst P Masson L Ismail SD Samsunder N Garrett N Mansoor LE. Cervicovaginal inflammation facilitates acquisition of less infectious HIV variants. Clin Infect Dis (2016) 64:79–82. doi: 10.1093/cid/ciw663 27694480 PMC5159604

[135] Masson L Passmore J-AS Liebenberg LJ Werner L Baxter C Arnold KB. Genital inflammation and the risk of HIV acquisition in women. Clin Infect Dis (2015) 61:260–9. doi: 10.1093/cid/civ298 PMC4565995 25900168

[136] Mohammadi A Bagherichimeh S Choi Y Fazel A Tevlin E Huibner S. Immune parameters of HIV susceptibility in the female genital tract before and after penile-vaginal sex. Commun Med (2022) 2:60. doi: 10.1038/s43856-022-00122-7 35637661 PMC9142516

[137] Annunziato F Cosmi L Liotta F Maggi E Romagnani S. Defining the human T helper 17 cell phenotype. Trends Immunol (2012) 33:505–12. doi: 10.1016/j.it.2012.05.004 22682163

[138] Lennard K Dabee S Barnabas SL Havyarimana E Blakney A Jaumdally SZ. Microbial composition predicts genital tract inflammation and persistent bacterial vaginosis in south African adolescent females. Infect Immun (2018) 86. doi: 10.1128/iai.00410-17 PMC5736802 29038128

[139] Alcaide ML Rodriguez VJ Brown MR Pallikkuth S Arheart K Martinez O. High levels of inflammatory cytokines in the reproductive tract of women with BV and engaging in intravaginal douching: A cross-sectional study of participants in the women interagency HIV study. AIDS Res Hum Retrov (2017) 33:309–17. doi: 10.1089/aid.2016.0187 PMC5372759 27897054

[140] McClelland RS Lingappa JR Srinivasan S Kinuthia J John-Stewart GC Jaoko W. Evaluation of the association between the concentrations of key vaginal bacteria and the increased risk of HIV acquisition in African women from five cohorts: a nested case-control study. Lancet Infect Dis (2018) 18:554–64. doi: 10.1016/s1473-3099(18)30058-6 PMC6445552 29396006

[141] Ghosh M Shen Z Fahey JV Cu-Uvin S Mayer K Wira CR. Trappin-2/Elafin: a novel innate anti-human immunodeficiency virus-1 molecule of the human female reproductive tract. Immunology (2010) 129:207–19. doi: 10.1111/j.1365-2567.2009.03165.x PMC2814463 19824918

[142] Shukair SA Allen SA Cianci GC Stieh DJ Anderson MR Baig SM. Human cervicovaginal mucus contains an activity that hinders HIV-1 movement. Mucosal Immunol (2013) 6:427–34. doi: 10.1038/mi.2012.87 PMC3732193 22990624

[143] Drannik AG Nag K Yao X-D Henrick BM Ball TB Plummer FA. Anti-HIV-1 activity of elafin depends on its nuclear localization and altered innate immune activation in female genital epithelial cells. PloS One (2012) 7:e52738. doi: 10.1371/journal.pone.0052738 23300756 PMC3531372

[144] Iqbal SM Ball TB Levinson P Maranan L Jaoko W Wachihi C. Elevated elafin/trappin-2 in the female genital tract is associated with protection against HIV acquisition. Aids (2009) 23:1669–77. doi: 10.1097/qad.0b013e32832ea643 19553806

[145] Wang Z Shang H Jiang Y. Chemokines and chemokine receptors: Accomplices for human immunodeficiency virus infection and latency. Front Immunol (2017) 8:1274. doi: 10.3389/fimmu.2017.01274 29085362 PMC5650658

[146] Lajoie J Poudrier J Loembe MM Guédou F Leblond F Labbé A-C. Chemokine expression patterns in the systemic and genital tract compartments are associated with HIV-1 infection in women from Benin. J Clin Immunol (2010) 30:90–8. doi: 10.1007/s10875-009-9343-3 19898927

[147] Kwaa AKR Talana CAG Blankson JN. Interferon alpha enhances NK cell function and the suppressive capacity of HIV-specific CD8 + T cells. J Virol (2019) 93 (3) :e01541-18. doi: 10.1128/jvi.01541-18 30404799 PMC6340025

[148] Chen K Huang J Zhang C Huang S Nunnari G Wang F. Alpha interferon potently enhances the anti-human immunodeficiency virus type 1 activity of APOBEC3G in resting primary CD4 T cells. J Virol (2006) 80:7645–57. doi: 10.1128/jvi.00206-06 PMC1563726 16840343

[149] Lai SK Hida K Shukair S Wang Y-Y Figueiredo A Cone R. Human immunodeficiency virus type 1 is trapped by acidic but not by neutralized human cervicovaginal mucus. J Virol (2009) 83:11196–200. doi: 10.1128/jvi.01899-08 PMC2772788 19692470

[150] Münch J Rücker E Ständker L Adermann K Goffinet C Schindler M. Semen-derived amyloid fibrils drastically enhance HIV infection. Cell (2007) 131:1059–71. doi: 10.1016/j.cell.2007.10.014 18083097

[151] Barr FD Ochsenbauer C Wira CR Rodriguez-Garcia M. Neutrophil extracellular traps prevent HIV infection in the female genital tract. Mucosal Immunol (2018) 11:1420–8. doi: 10.1038/s41385-018-0045-0 PMC6162173 29875403

[152] Hensley-McBain T Klatt NR. The dual role of neutrophils in HIV infection. Curr Hiv-aids Rep (2018) 15:1–10. doi: 10.1007/s11904-018-0370-7 29516266 PMC6086572

[153] Stieh DJ Matias E Xu H Fought AJ Blanchard JL Marx PA. Th17 cells are preferentially infected very early after vaginal transmission of SIV in macaques. Cell Host Microbe (2016) 19:529–40. doi: 10.1016/j.chom.2016.03.005 PMC4841252 27078070

[154] Rodriguez-Garcia M Shen Z Barr FD Boesch AW Ackerman ME Kappes JC. Dendritic cells from the human female reproductive tract rapidly capture and respond to HIV. Mucosal Immunol (2017) 10:531–44. doi: 10.1038/mi.2016.72 PMC5332537 27579858

[155] Wu L KewalRamani VN. Dendritic-cell interactions with HIV: infection and viral dissemination. Nat Rev Immunol (2006) 6:859–68. doi: 10.1038/nri1960 PMC1796806 17063186

[156] Cho S Knox KS Kohli LM He JJ Exley MA Wilson SB. Impaired cell surface expression of human CD1d by the formation of an HIV-1 Nef/CD1d complex. Virology (2005) 337:242–52. doi: 10.1016/j.virol.2005.04.020 15916790

[157] Chen N McCarthy C Drakesmith H Li D Cerundolo V McMichael AJ. HIV-1 down-regulates the expression of CD1d *via* nef. Eur J Immunol (2006) 36:278–86. doi: 10.1002/eji.200535487 16385629

[158] McKinnon LR Achilles SL Bradshaw CS Burgener A Crucitti T Fredricks DN. The evolving facets of bacterial vaginosis: Implications for HIV transmission. AIDS Res Hum Retrov (2019) 35:219–28. doi: 10.1089/aid.2018.0304 PMC6434601 30638028

[159] Kaambo E Africa C Chambuso R Passmore J-AS. Vaginal microbiomes associated with aerobic vaginitis and bacterial vaginosis. Front Public Heal (2018) 6:78. doi: 10.3389/fpubh.2018.00078 PMC5879096 29632854

[160] Gao E-K Lo D Cheney R Kanagawa O Sprent J. Abnormal differentiation of thymocytes in mice treated with cyclosporin a. Nature (1988) 336:176–9. doi: 10.1038/336176a0 2972933

[161] Muhleisen AL Herbst-Kralovetz MM. Menopause and the vaginal microbiome. Maturitas (2016) 91:42–50. doi: 10.1016/j.maturitas.2016.05.015 27451320

[162] Yamamoto T Zhou X Williams CJ Hochwalt A Forney LJ. Bacterial populations in the vaginas of healthy adolescent women. J Pediatr Adol Gynec (2009) 22:11–8. doi: 10.1016/j.jpag.2008.01.073 19232297

[163] Eschenbach DA Thwin SS Patton DL Hooton TM Stapleton AE Agnew K. Influence of the normal menstrual cycle on vaginal tissue, discharge, and microflora. Clin Infect Dis (2000) 30:901–7. doi: 10.1086/313818 10852812

[164] Petrova MI Broek Md Balzarini J Vanderleyden J Lebeer S. Vaginal microbiota and its role in HIV transmission and infection. FEMS Microbiol Rev (2013) 37:762–92. doi: 10.1111/1574-6976.12029 23789590

[165] Pavlova SI Kilic AO Kilic SS So J-S Nader-Macias ME Simoes JA. Genetic diversity of vaginal lactobacilli from women in different countries based on 16S rRNA gene sequences. J Appl Microbiol (2002) 92:451–9. doi: 10.1046/j.1365-2672.2002.01547.x 11872120

[166] Cheu RK Gustin AT Lee C Schifanella L Miller CJ Ha A. Impact of vaginal microbiome communities on HIV antiretroviral-based pre-exposure prophylaxis (PrEP) drug metabolism. PloS Pathog (2020) 16:e1009024. doi: 10.1371/journal.ppat.1009024 33270801 PMC7714160

[167] Onderdonk AB Delaney ML Fichorova RN. The human microbiome during bacterial vaginosis. Clin Microbiol Rev (2016) 29:223–38. doi: 10.1128/cmr.00075-15 PMC4786887 26864580

[168] Srinivasan S Hoffman NG Morgan MT Matsen FA Fiedler TL Hall RW. Bacterial communities in women with bacterial vaginosis: High resolution phylogenetic analyses reveal relationships of microbiota to clinical criteria. PloS One (2012) 7:e37818. doi: 10.1371/journal.pone.0037818 22719852 PMC3377712

[169] Jespers V Crucitti T Menten J Verhelst R Mwaura M Mandaliya K. Prevalence and correlates of bacterial vaginosis in different Sub-populations of women in Sub-Saharan Africa: A cross-sectional study. PloS One (2014) 9:e109670. doi: 10.1371/journal.pone.0109670 25289640 PMC4188821

[170] Martin HL Richardson BA Nyange PM Lavreys L Hillier SL Chohan B. Vaginal lactobacilli, microbial flora, and risk of human immunodeficiency virus type 1 and sexually transmitted disease acquisition. J Infect Dis (1999) 180:1863–8. doi: 10.1086/315127 10558942

[171] Martín R Soberón N Escobedo S Suárez JE. Bacteriophage induction versus vaginal homeostasis: role of H(2)O(2) in the selection of lactobacillus defective prophages. Int Microbiol Off J Span Soc Microbiol (2009) 12:131–6. 19784933

[172] Mitchell C Balkus JE Fredricks D Liu C McKernan-Mullin J Frenkel LM. Interaction between lactobacilli, bacterial vaginosis-associated bacteria, and HIV type 1 RNA and DNA genital shedding in U.S. and Kenyan women. AIDS Res Hum Retrov (2013) 29:13–9. doi: 10.1089/aid.2012.0187 PMC3537306 23020644

[173] Spear GT Gilbert D Landay AL Zariffard R French AL Patel P. Pyrosequencing of the genital microbiotas of HIV-seropositive and -seronegative women reveals lactobacillus iners as the predominant lactobacillus species. Appl Environ Microb (2011) 77:378–81. doi: 10.1128/aem.00973-10 PMC3019699 21075899

[174] Balkus JE Mitchell C Agnew K Liu C Fiedler T Cohn SE. Detection of hydrogen peroxide-producing lactobacillus species in the vagina: a comparison of culture and quantitative PCR among HIV-1 seropositive women. BMC Infect Dis (2012) 12:188. doi: 10.1186/1471-2334-12-188 22889380 PMC3462152

[175] Gosmann C Anahtar MN Handley SA Farcasanu M Abu-Ali G Bowman BA. Lactobacillus-deficient cervicovaginal bacterial communities are associated with increased HIV acquisition in young south African women. Immunity (2017) 46:29–37. doi: 10.1016/j.immuni.2016.12.013 28087240 PMC5270628

[176] Anahtar MN Byrne EH Doherty KE Bowman BA Yamamoto HS Soumillon M. Cervicovaginal bacteria are a major modulator of host inflammatory responses in the female genital tract. Immunity (2015) 42:965–76. doi: 10.1016/j.immuni.2015.04.019 PMC4461369 25992865

[177] Klatt NR Cheu R Birse K Zevin AS Perner M Noël-Romas L. Vaginal bacteria modify HIV tenofovir microbicide efficacy in African women. Science (2017) 356:938–45. doi: 10.1126/science.aai9383 28572388

[178] Price JT Vwalika B France M Ravel J Ma B Mwape H. HIV-Associated vaginal microbiome and inflammation predict spontaneous preterm birth in Zambia. Sci Rep-uk (2022) 12:8573. doi: 10.1038/s41598-022-12424-w PMC9123167 35595739

[179] Dieu-Nosjean M Vicari A Lebecque S Caux C. Regulation of dendritic cell trafficking: a process that involves the participation of selective chemokines. J Leukocyte Biol (1999) 66:252–62. doi: 10.1002/jlb.66.2.252 10449163

[180] Wira CR Fahey JV Sentman CL Pioli PA Shen L. Innate and adaptive immunity in female genital tract: cellular responses and interactions. Immunol Rev (2005) 206:306–35. doi: 10.1111/j.0105-2896.2005.00287.x 16048557

[181] Al-Harthi L Spear GT Hashemi FB Landay A Sha BE Roebuck KA. A human immunodeficiency virus (HIV)-inducing factor from the female genital tract activates HIV-1 gene expression through the κB enhancer. J Infect Dis (1998) 178:1343–51. doi: 10.1086/314444 9780254

[182] Aldunate M Tyssen D Johnson A Zakir T Sonza S Moench T. Vaginal concentrations of lactic acid potently inactivate HIV. J Antimicrob Chemoth (2013) 68:2015–25. doi: 10.1093/jac/dkt156 PMC3743514 23657804

[183] Mirmonsef P Gilbert D Veazey RS Wang J Kendrick SR Spear GT. A comparison of lower genital tract glycogen and lactic acid levels in women and macaques: Implications for HIV and SIV susceptibility. AIDS Res Hum Retrov (2012) 28:76–81. doi: 10.1089/aid.2011.0071 PMC3251838 21595610

[184] Borgdorff H Gautam R Armstrong SD Xia D Ndayisaba GF Teijlingen NHv. Cervicovaginal microbiome dysbiosis is associated with proteome changes related to alterations of the cervicovaginal mucosal barrier. Mucosal Immunol (2016) 9:621–33. doi: 10.1038/mi.2015.86 26349657

[185] Nunn KL Wang Y-Y Harit D Humphrys MS Ma B Cone R. Enhanced trapping of HIV-1 by human cervicovaginal mucus is associated with lactobacillus crispatus-dominant microbiota. Mbio (2015) 6:e01084–15. doi: 10.1128/mbio.01084-15 PMC4611035 26443453

[186] Spear GT Sikaroodi M Zariffard MR Landay AL French AL Gillevet PM. Comparison of the diversity of the vaginal microbiota in HIV-infected and HIV-uninfected women with or without bacterial vaginosis. J Infect Dis (2008) 198:1131–40. doi: 10.1086/591942 PMC2800037 18717638

[187] Gelber SE Aguilar JL Lewis KLT Ratner AJ. Functional and phylogenetic characterization of vaginolysin, the human-specific cytolysin from gardnerella vaginalis. J Bacteriol (2008) 190:3896–903. doi: 10.1128/jb.01965-07 PMC2395025 18390664

[188] Africa CWJ Nel J Stemmet M. Anaerobes and bacterial vaginosis in pregnancy: Virulence factors contributing to vaginal colonisation. Int J Environ Res Pu (2014) 11:6979–7000. doi: 10.3390/ijerph110706979 PMC4113856 25014248

[189] Kim S Ha M Choi E Choi J Choi I. Nitric oxide production and inducible nitric oxide synthase expression induced by prevotella nigrescens lipopolysaccharide. FEMS Immunol Med Microbiol (2005) 43:51–8. doi: 10.1016/j.femsim.2004.07.001 15607636

[190] Clottey C Dallabetta G. Sexually transmitted diseases and human immunodeficiency virus. epidemiologic synergy? Infect Dis Clin N Am (1993) 7:753–70. doi: 10.1016/S0891-5520(20)30558-4 8106728

[191] Royce RA Seña A Cates W Cohen MS. Sexual transmission of HIV. New Engl J Med (1997) 336:1072–8. doi: 10.1056/nejm199704103361507 9091805

[192] Minkoff HL Eisenberger-Matityahu D Feldman J Burk R Clarke L. Prevalence and incidence of gynecologic disorders among women infected with human immunodeficiency virus. Am J Obstet Gynecol (1999) 180:824–36. doi: 10.1016/s0002-9378(99)70653-8 10203650

[193] Greenblatt RM Bacchetti P Barkan S Augenbraun M Silver S Delapenha R. Lower genital tract infections among HIV-infected and high-risk uninfected women. Sex Transm Dis (1999) 26:143–51. doi: 10.1097/00007435-199903000-00004 10100771

[194] Burns DN Tuomala R Chang B-H Hershow R Minkoff H Rodriguez E. Vaginal colonization or infection with candida albicans in human immunodeficiency virus-infected women during pregnancy and during the postpartum period. Clin Infect Dis (1997) 24:201–10. doi: 10.1093/clinids/24.2.201 9114148

[195] Imam N Carpenter CCJ Mayer KH Fisher A Stein M Danforth SB. Hierarchical pattern of mucosal candida infections in HIV-seropositive women. Am J Med (1990) 89:142–6. doi: 10.1016/0002-9343(90)90291-k 1974383

[196] Schuman P Sobel JD Ohmit SE Mayer KH Carpenter CCJ Rompalo A. Mucosal candidal colonization and candidiasis in women with or at risk for human immunodeficiency virus infection. Clin Infect Dis (1998) 27:1161–7. doi: 10.1086/514979 9827263

[197] Hester RA Kennedy SB. Candida infection as a risk factor for HIV transmission. J Women’s Heal (2003) 12:487–94. doi: 10.1089/154099903766651612 12869296

[198] Hummelen R Fernandes AD Macklaim JM Dickson RJ Changalucha J Gloor GB. Deep sequencing of the vaginal microbiota of women with HIV. PloS One (2010) 5:e12078. doi: 10.1371/journal.pone.0012078 20711427 PMC2920804

[199] Sobel JD. Vulvovaginal candidiasis: a comparison of HIV-positive and -negative women. Int J Std AIDS (2002) 13:358–62. doi: 10.1258/095646202760029741 12015006

[200] Moodley P Connolly C Sturm AW. Interrelationships among human immunodeficiency virus type 1 infection, bacterial vaginosis, trichomoniasis, and the presence of yeasts. J Infect Dis (2002) 185:69–73. doi: 10.1086/338027 11756983

[201] Fidel PL. Vaginal candidiasis: Review and role of local mucosal immunity. AIDS Patient Care St (1998) 12:359–66. doi: 10.1089/apc.1998.12.359 11361971

[202] Chaisilwattana P Chuachoowong R Siriwasin W Bhadrakom C Mangclaviraj Y Young Nl. Chlamydial and gonococcal cervicitis in HIV-seropositive and HIV-seronegative pregnant women in Bangkok. Sex Transm Dis (1997) 24:495–502. doi: 10.1097/00007435-199710000-00001 9339966

[203] Kreiss J Willerford DM Hensel M Emonyi W Plummer F Ndinya-Achola J. Association between cervical inflammation and cervical shedding of human immunodeficiency virus DNA. J Infect Dis (1994) 170:1597–601. doi: 10.1093/infdis/170.6.1597 7996003

[204] Maier JA Bergman A Ross MG. Acquired immunodeficiency syndrome manifested by chronic primary genital herpes. Am J Obstet Gynecol (1986) 155:756–8. doi: 10.1016/s0002-9378(86)80014-x 3766631

[205] Fennema JSA Ameijden EJCv Coutinho RA Hoek A(J)AR. HIV, Sexually transmitted diseases and gynaecologic disorders in women. Aids (1995) 9:1071–8. doi: 10.1097/00002030-199509000-00014 8527081

[206] Lemos MP Lama JR Karuna ST Fong Y Montano SM Ganoza C. The inner foreskin of healthy males at risk of HIV infection harbors epithelial CD4+ CCR5+ cells and has features of an inflamed epidermal barrier. PloS One (2014) 9:e108954. doi: 10.1371/journal.pone.0108954 25268493 PMC4182607

[207] McCoombe SG Short RV. Potential HIV-1 target cells in the human penis. Aids (2006) 20:1491–5. doi: 10.1097/01.aids.0000237364.11123.98 16847403

[208] Mills E Cooper C Anema A Guyatt G. Male Circumcision for the prevention of heterosexually acquired HIV infection: a meta-analysis of randomized trials involving 11 050 men*. HIV Med (2008) 9:332–5. doi: 10.1111/j.1468-1293.2008.00596.x 18705758

[209] Price LB Liu CM Johnson KE Aziz M Lau MK Bowers J. The effects of circumcision on the penis microbiome. PloS One (2010) 5:e8422. doi: 10.1371/journal.pone.0008422 20066050 PMC2798966

[210] Onywera H Williamson A-L Cozzuto L Bonnin S Mbulawa ZZA Coetzee D. The penile microbiota of black south African men: relationship with human papillomavirus and HIV infection. BMC Microbiol (2020) 20:78. doi: 10.1186/s12866-020-01759-x 32252632 PMC7137192

[211] Liu CM Hungate BA Tobian AAR Serwadda D Ravel J Lester R. Male Circumcision significantly reduces prevalence and load of genital anaerobic bacteria. Mbio (2013) 4:e00076–13. doi: 10.1128/mbio.00076-13 PMC3634604 23592260

[212] Liu CM Prodger JL Tobian AAR Abraham AG Kigozi G Hungate BA. Penile anaerobic dysbiosis as a risk factor for HIV infection. Mbio (2017) 8:e00996–17. doi: 10.1128/mbio.00996-17 PMC5527312 28743816

[213] Fleiss PM Hodges FM Howe RSV. Immunological functions of the human prepuce. Sex Transm Infect (1998) 74:364. doi: 10.1136/sti.74.5.364 10195034 PMC1758142

[214] Anderson D Politch JA Pudney J. HIV Infection and immune defense of the penis. Am J Reprod Immunol (2011) 65:220–9. doi: 10.1111/j.1600-0897.2010.00941.x PMC3076079 21214659

[215] Prodger JL Gray R Kigozi G Nalugoda F Galiwango R Hirbod T. Foreskin T-cell subsets differ substantially from blood with respect to HIV co-receptor expression, inflammatory profile, and memory status. Mucosal Immunol (2012) 5:121–8. doi: 10.1038/mi.2011.56 PMC3288185 22089029

[216] Prodger JL Kaul R. The biology of how circumcision reduces HIV susceptibility: broader implications for the prevention field. AIDS Res Ther (2017) 14:49. doi: 10.1186/s12981-017-0167-6 28893286 PMC5594533

[217] Patil S Majumdar B Sarode SC Sarode GS Awan KH. Oropharyngeal candidosis in HIV-infected patients–an update. Front Microbiol (2018) 9:980. doi: 10.3389/fmicb.2018.00980 29867882 PMC5962761

[218] Winter AJ Taylor S Workman J White D Ross JD Swan AV. Asymptomatic urethritis and detection of HIV-1 RNA in seminal plasma. Sex Transm Infect (1999) 75:261. doi: 10.1136/sti.75.4.261 10615314 PMC1758225

[219] Pérez-Soto E Fernández-Martínez E Oros-Pantoja R Medel-Flores O Miranda-Covarrubias JC Sánchez-Monroy V. Proinflammatory and oxidative stress states induced by human papillomavirus and chlamydia trachomatis coinfection affect sperm quality in asymptomatic infertile men. Medicina (2021) 57:862. doi: 10.3390/medicina57090862 34577786 PMC8466842

[220] Gray CM O’Hagan KL Lorenzo-Redondo R Olivier AJ Amu S Chigorimbo-Murefu N. Impact of chemokine c–c ligand 27, foreskin anatomy and sexually transmitted infections on HIV-1 target cell availability in adolescent south African males. Mucosal Immunol (2020) 13:118–27. doi: 10.1038/s41385-019-0209-6 PMC6914668 31619762

[221] Gianella S Strain MC Rought SE Vargas MV Little SJ Richman DD. Associations between virologic and immunologic dynamics in blood and in the Male genital tract. J Virol (2012) 86:1307–15. doi: 10.1128/jvi.06077-11 PMC3264378 22114342

[222] Freeman EE Weiss HA Glynn JR Cross PL Whitworth JA Hayes RJ. Herpes simplex virus 2 infection increases HIV acquisition in men and women&colon; systematic review and meta-analysis of longitudinal studies. Aids (2006) 20:73–83. doi: 10.1097/01.aids.0000198081.09337.a7 16327322

[223] Perre PVd Segondy M Foulongne V Ouedraogo A Konate I Huraux J-M. Herpes simplex virus and HIV-1: deciphering viral synergy. Lancet Infect Dis (2008) 8:490–7. doi: 10.1016/s1473-3099(08)70181-6 18652995

[224] Prevention c for DC and. CDC HIV risk behaviors (2022). Available at: https://www.cdc.gov/hiv/risk/estimates/riskbehaviors.html (Accessed November 15, 2022).

[225] Chenine AL Siddappa NB Kramer VG Sciaranghella G Rasmussen RA Lee SJ. Relative transmissibility of an R5 clade c simian- human immunodeficiency virus across different mucosae in macaques parallels the relative risks of sexual HIV-1 transmission in humans *via* different routes. J Infect Dis (2010) 201:1155–63. doi: 10.1086/651274 PMC2838976 20214475

[226] Prevention c for DC and. morbidity and mortality weekly report (MMWR) (2013). Available at: https://www.cdc.gov/mmwr/preview/mmwrhtml/su6203a19.htm (Accessed November 15, 2022).

[227] Shakirzyanova M Tsai L Ren W Gettie A Blanchard J Cheng-Mayer C. Pathogenic consequences of vaginal infection with CCR5-tropic simian-human immunodeficiency virus SHIV SF162P3N. J Virol (2012) 86:9432–42. doi: 10.1128/jvi.00852-12 PMC3416103 22740397

[228] Tebit DM Ndembi N Weinberg A Quiñones-Mateu ME. Mucosal transmission of human immunodeficiency virus. Curr HIV Res (2011) 10:3–8. doi: 10.2174/157016212799304689 PMC3744389 22264040

[229] Keele BF Estes JD. Barriers to mucosal transmission of immunodeficiency viruses. Blood (2011) 118:839–46. doi: 10.1182/blood-2010-12-325860 PMC3148165 21555745

[230] Carnathan DG Wetzel KS Yu J Lee ST Johnson BA Paiardini M. Activated CD4+CCR5+ T cells in the rectum predict increased SIV acquisition in SIVGag/Tat-vaccinated rhesus macaques. P Natl Acad Sci USA (2014) 112:518–23. doi: 10.1073/pnas.1407466112 PMC4299179 25550504

[231] McElrath MJ Smythe K Randolph-Habecker J Melton KR Goodpaster TA Hughes SM. Comprehensive assessment of HIV target cells in the distal human gut suggests increasing HIV susceptibility toward the anus. Jaids J Acquir Immune Defic Syndromes (2013) 63:263–71. doi: 10.1097/qai.0b013e3182898392 PMC3683090 23392465

[232] Leoni G Neumann P-A Sumagin R Denning TL Nusrat A. Wound repair: role of immune-epithelial interactions. Mucosal Immunol (2014) 8:959–68. doi: 10.1038/mi.2015.63 PMC4916915 26174765

[233] Kelley CF Kraft CS de MTJ Duphare C Lee H-W Yang J. The rectal mucosa and condomless receptive anal intercourse in HIV-negative MSM: implications for HIV transmission and prevention. Mucosal Immunol (2017) 10:996–1007. doi: 10.1038/mi.2016.97 27848950 PMC5433931

[234] Cory TJ Schacker TW Stevenson M Fletcher CV. Overcoming pharmacologic sanctuaries. Curr Opin HIV AIDS (2013) 8:190–5. doi: 10.1097/coh.0b013e32835fc68a PMC3677586 23454865

[235] Sattentau QJ Stevenson M. Macrophages and HIV-1: An unhealthy constellation. Cell Host Microbe (2016) 19:304–10. doi: 10.1016/j.chom.2016.02.013 PMC5453177 26962941

[236] Brown D Mattapallil JJ. Gastrointestinal tract and the mucosal macrophage reservoir in HIV infection. Clin Vaccine Immunol (2014) 21:1469–73. doi: 10.1128/cvi.00518-14 PMC4248758 25185575

[237] Gurney KB Elliott J Nassanian H Song C Soilleux E McGowan I. Binding and transfer of human immunodeficiency virus by DC-SIGN + cells in human rectal mucosa. J Virol (2005) 79:5762–73. doi: 10.1128/jvi.79.9.5762-5773.2005 PMC1082722 15827191

[238] Kane M Yadav SS Bitzegeio J Kutluay SB Zang T Wilson SJ. MX2 is an interferon-induced inhibitor of HIV-1 infection. Nature (2013) 502:563–6. doi: 10.1038/nature12653 PMC3912734 24121441

[239] Liu Z Pan Q Ding S Qian J Xu F Zhou J. The interferon-inducible MxB protein inhibits HIV-1 infection. Cell Host Microbe (2013) 14:398–410. doi: 10.1016/j.chom.2013.08.015 24055605

[240] Maric D Grimm WA Greco N McRaven MD Fought AJ Veazey RS. Th17 T cells and immature dendritic cells are the preferential initial targets after rectal challenge with a simian immunodeficiency virus-based replication-defective dual-reporter vector. J Virol (2021) 95:e00707–21. doi: 10.1128/jvi.00707-21 PMC8428389 34287053

[241] Taylor HE Calantone N Lichon D Hudson H Clerc I Campbell EM. mTOR overcomes multiple metabolic restrictions to enable HIV-1 reverse transcription and intracellular transport. Cell Rep (2020) 31:107810. doi: 10.1016/j.celrep.2020.107810 32579936 PMC7389891

[242] Pan X Baldauf H-M Keppler OT Fackler OT. Restrictions to HIV-1 replication in resting CD4+ T lymphocytes. Cell Res (2013) 23:876–85. doi: 10.1038/cr.2013.74 PMC3698640 23732522

[243] Wilflingseder D Schroll A Hackl H Gallasch R Frampton D Lass-Flörl C. Immediate T-helper 17 polarization upon triggering CD11b/c on HIV-exposed dendritic cells. J Infect Dis (2015) 212:44–56. doi: 10.1093/infdis/jiv014 25583169

[244] Saifuddin M Parker CJ Peeples ME Gorny MK Zolla-Pazner S Ghassemi M. Role of virion-associated glycosylphosphatidylinositol-linked proteins CD55 and CD59 in complement resistance of cell line-derived and primary isolates of HIV-1. J Exp Med (1995) 182:501–9. doi: 10.1084/jem.182.2.501 PMC2192116 7543140

[245] Stoiber H Banki Z Wilflingseder D Dierich MP. Complement–HIV interactions during all steps of viral pathogenesis. Vaccine (2008) 26:3046–54. doi: 10.1016/j.vaccine.2007.12.003 18191309

[246] Bhattacharya P Ellegård R Khalid M Svanberg C Govender M Keita ÅV. Complement opsonization of HIV affects primary infection of human colorectal mucosa and subsequent activation of T cells. Elife (2020) 9:e57869. doi: 10.7554/elife.57869 32876566 PMC7492089

[247] Bouhlal H Chomont N Réquena M Nasreddine N Saidi H Legoff J. Opsonization of HIV with complement enhances infection of dendritic cells and viral transfer to CD4 T cells in a CR3 and DC-SIGN-Dependent manner. J Immunol (2007) 178:1086–95. doi: 10.4049/jimmunol.178.2.1086 17202372

[248] Nijmeijer BM Bermejo-Jambrina M Kaptein TM Ribeiro CMS Wilflingseder D Geijtenbeek TBH. HIV-1 subverts the complement system in semen to enhance viral transmission. Mucosal Immunol (2021) 14:743–50. doi: 10.1038/s41385-021-00376-9 PMC8075950 33568786

[249] Svanberg C Ellegård R Crisci E Khalid M Wodlin NB Svenvik M. Complement-opsonized HIV modulates pathways involved in infection of cervical mucosal tissues: A transcriptomic and proteomic study. Front Immunol (2021) 12:625649. doi: 10.3389/fimmu.2021.625649 34093520 PMC8173031

[250] Kovach R Goldberg S. Interventional treatment in evolving myocardial infarction. Cardiology (1989) 76:158–66. doi: 10.1159/000174486 2525956

[251] Ellegård R Crisci E Andersson J Shankar EM Nyström S Hinkula J. Impaired NK cell activation and chemotaxis toward dendritic cells exposed to complement-opsonized HIV-1. J Immunol (2015) 195:1698–704. doi: 10.4049/jimmunol.1500618 PMC4528080 26157174

[252] Denny JE Powell WL Schmidt NW. Local and long-distance calling: Conversations between the gut microbiota and intra- and extra-gastrointestinal tract infections. Front Cell Infect Mi (2016) 6:41. doi: 10.3389/fcimb.2016.00041 PMC4826874 27148490

[253] Lozupone CA Li M Campbell TB Flores SC Linderman D Gebert MJ. Alterations in the gut microbiota associated with HIV-1 infection. Cell Host Microbe (2013) 14:329–39. doi: 10.1016/j.chom.2013.08.006 PMC3864811 24034618

[254] Sui Y Dzutsev A Venzon D Frey B Thovarai V Trinchieri G. Influence of gut microbiome on mucosal immune activation and SHIV viral transmission in naive macaques. Mucosal Immunol (2018) 11:1219–29. doi: 10.1038/s41385-018-0029-0 PMC6030500 29858581

[255] Noguera-Julian M Rocafort M Guillén Y Rivera J Casadellà M Nowak P. Gut microbiota linked to sexual preference and HIV infection. Ebiomedicine (2016) 5:135–46. doi: 10.1016/j.ebiom.2016.01.032 PMC4816837 27077120

[256] Ceccarani C Marangoni A Severgnini M Camboni T Laghi L Gaspari V. Rectal microbiota associated with chlamydia trachomatis and neisseria gonorrhoeae infections in men having sex with other men. Front Cell Infect Mi (2019) 9:358. doi: 10.3389/fcimb.2019.00358 PMC6813206 31681634

[257] Barbee LA Soge OO Katz DA Dombrowski JC Holmes KK Golden MR. Increases in neisseria gonorrhoeae with reduced susceptibility to azithromycin among men who have sex with men in Seattle, king county, Washington, 2012–2016. Clin Infect Dis (2017) 66:712–8. doi: 10.1093/cid/cix898 PMC5848236 29045604

[258] Xu L Tudor D Bomsel M. The protective HIV-1 envelope gp41 antigen P1 acts as a mucosal adjuvant stimulating the innate immunity. Front Immunol (2021) 11:599278. doi: 10.3389/fimmu.2020.599278 33613520 PMC7886812

[259] Pavot V Rochereau N Lawrence P Girard MP Genin C Verrier B. Recent progress in HIV vaccines inducing mucosal immune responses. Aids (2014) 28:1701–18. doi: 10.1097/qad.0000000000000308 25009956

[260] Kozlowski PA Aldovini A. Mucosal vaccine approaches for prevention of HIV and SIV transmission. Curr Immunol Rev (2019) 15:102–22. doi: 10.2174/1573395514666180605092054 PMC6709706 31452652

[261] Moodie Z Janes H Huang Y. New clinical trial designs for HIV vaccine evaluation. Curr Opin HIV AIDS (2013) 8:437–42. doi: 10.1097/coh.0b013e328363d46a PMC3872029 23872613

[262] Astronomo RD Lemos MP Narpala SR Czartoski J Fleming LB Seaton KE. Rectal and vaginal tissue from intravenous VRC01 recipients show protection against *ex vivo* HIV-1 challenge. J Clin Invest (2021) 131 (16) :e146975. doi: 10.1172/jci146975 34166231 PMC8363291

[263] Lehner T Tao L Panagiotidi C Klavinskis LS Brookes R Hussain L. Mucosal model of genital immunization in male rhesus macaques with a recombinant simian immunodeficiency virus p27 antigen. J Virol (1994) 68:1624–32. doi: 10.1128/jvi.68.3.1624-1632.1994 PMC236620 8107223

[264] Vajdy M. Current efforts on generation of optimal immune responses against HIV through mucosal immunisations. Drugs R D (2006) 7:267–88. doi: 10.2165/00126839-200607050-00001 16922589

[265] Lycke N. Recent progress in mucosal vaccine development: potential and limitations. Nat Rev Immunol (2012) 12:592–605. doi: 10.1038/nri3251 22828912

[266] Kraan H Vrieling H Czerkinsky C Jiskoot W Kersten G Amorij J-P. Buccal and sublingual vaccine delivery. J Control Release (2014) 190:580–92. doi: 10.1016/j.jconrel.2014.05.060 PMC7114675 24911355

[267] Hervouet C Luci C Çuburu N Cremel M Bekri S Vimeux L. Sublingual immunization with an HIV subunit vaccine induces antibodies and cytotoxic T cells in the mouse female genital tract. Vaccine (2010) 28:5582–90. doi: 10.1016/j.vaccine.2010.06.033 20600505

[268] Jones AT Shen X Walter KL LaBranche CC Wyatt LS Tomaras GD. HIV-1 vaccination by needle-free oral injection induces strong mucosal immunity and protects against SHIV challenge. Nat Commun (2019) 10:798. doi: 10.1038/s41467-019-08739-4 30778066 PMC6379385

[269] Lewis DJM Wang Y Huo Z Giemza R Babaahmady K Rahman D. Effect of vaginal immunization with HIVgp140 and HSP70 on HIV-1 replication and innate and T cell adaptive immunity in women. J Virol (2014) 88:11648–57. doi: 10.1128/jvi.01621-14 PMC4178755 25008917

[270] Yoshizawa I Mizuochi T Ogata A Murakami M Yagita H Takahashi Y. Studies on the generation and maintenance of mucosal cytotoxic T lymphocytes against human immunodeficiency virus type 1 gag in mice. AIDS Res Hum Retrov (2003) 19:469–79. doi: 10.1089/088922203766774513 12882656

[271] Hamajima K Hoshino Y Xin K-Q Hayashi F Tadokoro K Okuda K. Systemic and mucosal immune responses in mice after rectal and vaginal immunization with HIV-DNA vaccine. Clin Immunol (2002) 102:12–8. doi: 10.1006/clim.2001.5141 11781062

[272] Trujillo-Vargas CM Mayer KD Bickert T Palmetshofer A Grunewald S Ramirez-Pineda JR. Vaccinations with T-helper type 1 directing adjuvants have different suppressive effects on the development of allergen-induced T-helper type 2 responses. Clin Exp Allergy (2005) 35:1003–13. doi: 10.1111/j.1365-2222.2005.02287.x 16120081

[273] Reikvam DH Meyer-Myklestad MH Trøseid M Stiksrud B. Probiotics to manage inflammation in HIV infection. Curr Opin Infect Dis (2020) 33:34–43. doi: 10.1097/qco.0000000000000612 31789692

[274] Klatt NR Broedlow C Osborn JM Gustin AT Dross S O’Connor MA. Effects of persistent modulation of intestinal microbiota on SIV/HIV vaccination in rhesus macaques. NPJ Vaccines (2021) 6:34. doi: 10.1038/s41541-021-00298-4 33707443 PMC7952719

[275] Borgognone A Noguera-Julian M Oriol B Noël-Romas L Ruiz-Riol M Guillén Y. Gut microbiome signatures linked to HIV-1 reservoir size and viremia control. Microbiome (2022) 10:59. doi: 10.1186/s40168-022-01247-6 35410461 PMC9004083

[276] Zhen A Peterson CW Carrillo MA Reddy SS Youn CS Lam BB. Long-term persistence and function of hematopoietic stem cell-derived chimeric antigen receptor T cells in a nonhuman primate model of HIV/AIDS. PloS Pathog (2017) 13:e1006753. doi: 10.1371/journal.ppat.1006753 29284044 PMC5746250

[277] Zhen A Carrillo MA Kitchen SG. Chimeric antigen receptor engineered stem cells: a novel HIV therapy. Immunotherapy (2017) 9:401–10. doi: 10.2217/imt-2016-0121 PMC5618937 28357916

[278] Yilmaz A Cui H Caligiuri MA Yu J. Chimeric antigen receptor-engineered natural killer cells for cancer immunotherapy. J Hematol Oncol (2020) 13:168. doi: 10.1186/s13045-020-00998-9 33287875 PMC7720606

[279] Nelson KN Hui Q Rimland D Xu K Freiberg MS Justice AC. Identification of HIV infection-related DNA methylation sites and advanced epigenetic aging in HIV-positive, treatment-naive U.S. veterans. Aids (2017) 31:571–5. doi: 10.1097/qad.0000000000001360 PMC5263111 27922854

[280] Rickabaugh TM Baxter RM Sehl M Sinsheimer JS Hultin PM Hultin LE. Acceleration of age-associated methylation patterns in HIV-1-Infected adults. PloS One (2015) 10:e0119201. doi: 10.1371/journal.pone.0119201 25807146 PMC4373843

[281] Zhang X Justice AC Hu Y Wang Z Zhao H Wang G. Epigenome-wide differential DNA methylation between HIV-infected and uninfected individuals. Epigenetics (2016) 11:750–60. doi: 10.1080/15592294.2016.1221569 PMC5094631 27672717

[282] Chen J Huang Y Hui Q Mathur R Gwinn M So-Armah K. Epigenetic associations with estimated glomerular filtration rate among men with human immunodeficiency virus infection. Clin Infect Dis (2019) 70:667–73. doi: 10.1093/cid/ciz240 PMC7319269 30893429

[283] Sundermann EE Hussain MA Moore DJ Horvath S Lin DTS Kobor MS. Inflammation-related genes are associated with epigenetic aging in HIV. J Neurovirol (2019) 25:853–65. doi: 10.1007/s13365-019-00777-4 PMC6923602 31286441

[284] Dye CK Corley MJ Li D Khadka VS Mitchell BI Sultana R. Comparative DNA methylomic analyses reveal potential origins of novel epigenetic biomarkers of insulin resistance in monocytes from virally suppressed HIV-infected adults. Clin Epigenet (2019) 11:95. doi: 10.1186/s13148-019-0694-1 PMC6599380 31253200

[285] Vaccari M Fourati S Gordon SN Brown DR Bissa M Schifanella L. HIV Vaccine candidate activation of hypoxia and the inflammasome in CD14+ monocytes is associated with a decreased risk of SIVmac251 acquisition. Nat Med (2018) 24:847–56. doi: 10.1038/s41591-018-0025-7 PMC5992093 29785023

[286] Jensen K Pena-Ponce MGd Piatak M Shoemaker R Oswald K Jacobs WR. Balancing trained immunity with persistent immune activation and the risk of simian immunodeficiency virus infection in infant macaques vaccinated with attenuated mycobacterium tuberculosis or mycobacterium bovis BCG vaccine. Clin Vaccine Immunol (2017) 24 (1) :e00360-16. doi: 10.1128/cvi.00360-16 27655885 PMC5216431

[287] Sugawara S Reeves RK Jost S. Learning to be elite: Lessons from HIV-1 controllers and animal models on trained innate immunity and virus suppression. Front Immunol (2022) 13:858383. doi: 10.3389/fimmu.2022.858383 35572502 PMC9094575

